# Disease‐Specific Determinants of Cardiovascular Nanomedicine: Mechanisms, Limitations, and Translational Opportunities

**DOI:** 10.1155/cdr/8263083

**Published:** 2026-11-06

**Authors:** Boopalan Sivanathan, Sharan Bobbala, Sundararajan Venkatesh

**Affiliations:** ^1^ Department of Physiology Pharmacology and Toxicology School of Medicine West Virginia University Morgantown West Virginia USA wvu.edu; ^2^ Department of Pharmaceutical Sciences School of Pharmacy West Virginia University Morgantown West Virginia USA wvu.edu

**Keywords:** cardiomyocyte targeting, drug delivery, heart disease, heart failure, nanoparticle

## Abstract

Cardiovascular disease (CVD) remains the leading cause of mortality worldwide, yet most therapies lack cardiovascular tissue‐ and cell‐specificity, limiting efficacy and increasing off‐target toxicity. Nanoparticle‐based drug delivery systems offer opportunities to enhance myocardial targeting, improve pharmacokinetics, and enable controlled or stimuli‐responsive release. However, despite extensive preclinical success, clinical translation of cardiovascular nanomedicine remains limited. A major gap is the insufficient integration of disease‐specific cardiac biology into nanoparticle design. Here, we propose that the success of cardiovascular nanomedicine depends on integrating nanoparticle physicochemical properties and disease‐specific microenvironmental conditions, including inflammation, vascular permeability, fibrosis, immune activation, and intracellular stress signaling. We provide a mechanistic and disease‐contextual framework to guide nanoparticle design across major cardiovascular conditions, including myocardial infarction, heart failure, atherosclerosis, and doxorubicin‐induced cardiotoxicity, while distinguishing cardiomyocyte‐directed delivery from vascular‐, immune‐cell‐, and stromal‐targeted strategies. Here, we critically evaluate current targeting strategies and major translation barriers associated with cell‐specific delivery, off‐target organ accumulation, preclinical models, manufacturing and scalability, and long‐term safety. This review shifts the paradigm from material‐centric to biology‐guided nanoparticle design and outlines actionable strategies to advance cardiovascular precision therapeutics toward clinical translation.

## 1. Introduction

Cardiovascular disease (CVD) remains the leading cause of mortality globally. The most recent Global Burden of Disease (GBD) 2023 analysis estimated 19.2 million CVD‐related deaths, 437 million disability‐adjusted life years (DALYs), and 626 million prevalent cases worldwide in 2023 [1]. Consistently, the World Health Organization (WHO) estimates that CVD accounted for approximately 19.8 million deaths in 2022, representing ~32% of all global deaths, with more than three‐quarters occurring in low‐ and middle‐income countries [2]. In addition to its substantial mortality and disability burden, CVD imposes major economic costs on healthcare systems and society. In the United States, the annual direct and indirect costs of CVD were estimated at approximately $414.7 billion in 2021–2022, including $223.2 billion in healthcare expenditures and $191.5 billion in productivity losses attributable to premature cardiovascular mortality [3]. Similarly, the economic burden of CVD across 25 European Union countries was estimated at approximately €169 billion annually, highlighting the substantial societal and healthcare costs associated with cardiovascular disorders [4]. CVD encompasses biologically distinct conditions, including myocardial infarction (MI), heart failure (HF), and atherosclerosis, while treatment‐related conditions such as doxorubicin (DOX)‐induced cardiotoxicity represent an additional and increasingly important form of cardiac injury. Within this overall burden, ischemic heart disease (IHD), which encompasses most MI‐related mortality, remains the leading cardiovascular cause of death and disability, whereas HF represents a heterogeneous downstream syndrome, atherosclerosis underlies much of ischemic CVD, and DOX‐induced cardiotoxicity constitutes a treatment‐associated cardiac burden that is not captured as an independent category in global GBD estimates. GBD 2023 reports approximately 8.91 million IHD deaths and 193 million IHD DALYs in 2023. These conditions differ substantially in their pathological microenvironments and therefore present different opportunities and barriers for targeted drug delivery [5, 6].

Although most drugs used today to treat CVDs act systemically, their central therapeutic limitation is that they are not disease‐ or heart‐ or cell‐type‐specific. Systemic administration exposes multiple organs, narrowing therapeutic indices and producing off‐target toxicities that cap dose and blunt efficacy at the site of disease [7]. Although current therapies improve cardiovascular function, they can be made more effective by delivering them to defined cardiac, vascular, or immune‐cell populations. Biological and physical barriers to reaching cardiomyocytes and other disease‐relevant cell populations, including rapid coronary blood flow and washout, endothelial and extracellular‐matrix barriers, and continuous myocardial contraction, further limit tissue exposure and retention [7]. Targeted delivery therefore offers the potential to achieve greater local therapeutic concentrations at lower doses while reducing systemic toxicity [8, 9].

Nanoparticle (NP)‐based drug delivery platforms offer a compelling approach to overcome these challenges. NPs are highly versatile, and their size, morphology, surface chemistry, and drug release kinetics can be tuned for site‐specific delivery. NPs can also enhance the solubility of hydrophobic drugs for easier administration, increase drug half‐life by prolonging systemic circulation, and improve myocardial drug retention through targeting [5]. Targeting can be achieved passively, without ligands, or actively, using ligands that recognize disease‐induced surface markers, or via biomimetic coatings that evade clearance and home to cardiac tissue [10]. Together, these features enable higher local drug concentrations in targeted cells with lower systemic exposure, potentially minimizing adverse effects while maximizing therapeutic impact [11, 12].

Most recent cardiovascular nanomedicine reviews [13, 14] have primarily emphasized NP composition, physicochemical properties, therapeutic cargo, or targeting modality. We acknowledge that these approaches provide valuable information on nanomaterial engineering; however, the disease‐specific microenvironment remains a critical determinant of NP design. For instance, MI, HF, atherosclerosis, and DOX cardiotoxicity provide fundamentally different biological environments. MI is characterized by acute inflammation, acidosis, oxidative stress, and transient vascular permeability; HF by impaired perfusion, extracellular matrix remodeling, and fibrosis; atherosclerosis by endothelial dysfunction and macrophage‐rich plaques; and DOX cardiotoxicity by cardiomyocyte oxidative and mitochondrial stress [15, 16]. These differences may directly influence NP accumulation, penetration, cellular uptake, and drug release and therefore guide the selection of particle size, surface charge, targeting ligand, cargo, and stimuli‐responsive release mechanisms.

In this review, we predominantly use a material‐centered classification and disease biology–guided framework while integrating the pathological microenvironment to define the delivery barrier and NP design. We evaluate how NP properties and targeting strategies can be matched to the distinct pathophysiology of MI, HF, atherosclerosis, and DOX‐induced cardiotoxicity, while explicitly distinguishing cardiomyocyte‐directed delivery from vascular‐, immune cell‐, and stromal‐targeted approaches. We further discuss emerging RNA‐encapsulating lipid NPs (LNPs) and extracellular vesicle (EV) platforms, organelle and multilevel targeting strategies, and critically evaluate the mechanistic, safety, manufacturing, regulatory, and clinical barriers to translation. By linking disease‐specific cardiovascular biology with NP engineering, this review aims to address a key gap in existing reviews and provide a rational framework for developing more precise and clinically translatable cardiovascular nanomedicines.

## 2. Types of NPs

NPs have emerged as versatile platforms for targeted drug delivery in CVD, overcoming long‐standing limitations such as poor aqueous solubility and cell permeability, rapid clearance, and systemic toxicity [17]. NPs for biomedical applications can be broadly defined as particles in any dimension with a measure < 1000 nm [18]. Engineered at the nanoscale (10^−9^ m), these systems can be tailored to enhance bioavailability while minimizing off‐target effects, representing a paradigm shift in delivering therapeutic agents to diseased cardiac tissue. In recent years, researchers have designed diverse NP formulations to diagnose and treat primary cardiac conditions, including atherosclerosis, MI, and HF, where precise delivery to disease‐relevant cardiovascular tissues and cell populations may improve therapeutic efficacy while limiting systemic exposure [19]. The unique physicochemical attributes of NPs, such as high surface area‐to‐volume ratio, tunable size and surface charge, and modifiable surface chemistry, allow them to interact efficiently with biological systems and cross physiological barriers that limit conventional drug delivery. Key properties such as biocompatibility, biodegradability, and aqueous dispersibility make NPs particularly suitable for biomedical applications [20–22]. These materials can encapsulate both hydrophilic and hydrophobic drugs, protect molecules from in vivo degradation, and provide controlled or sustained release profiles. Notably, NPs increase drug systemic circulation time and half‐life, improving bioavailability and broadening the therapeutic index of many cardiovascular drugs with poor pharmacokinetics [23, 24].

Importantly, a NP platform’s suitability for cardiovascular therapy depends not only on its material properties but also on the microenvironment in which it must function. For example, transient vascular permeability and inflammation following MI may facilitate tissue accumulation, whereas reduced perfusion and fibrosis in chronic HF can restrict NP penetration. In atherosclerosis, endothelial dysfunction and macrophage‐rich plaques provide vascular and immune‐cell targets, whereas DOX‐induced cardiotoxicity may require efficient cardiomyocyte uptake and intracellular or mitochondrial delivery. Thus, the properties of each NP class should be considered in relation to the biological barriers and therapeutic targets of the specific CVD.

The major organic, inorganic, and hybrid NP platforms are summarized below, with emphasis on the properties that influence their suitability for disease‐specific cardiovascular delivery. Table 1 compares their major physicochemical characteristics, cargo suitability, safety considerations, routes of administration, and translational maturity.

**Table 1:** Comparative characteristics and translational considerations of major nanoparticle platforms for cardiovascular drug delivery.

Platform	Typical size range^a^	Best‐suited cargo	Route	Biodegradability	Key safety concern	Cardiovascular translational maturity
Lipid nanoparticles (LNPs)	~60–150 nm	mRNA, siRNA, other nucleic acids; selected small molecules	IV; SC; IM	High	Predominant liver uptake, innate immune activation, anti‐PEG responses, and formulation‐dependent reactogenicity	Preclinical for cardiac targeting/RNA delivery; clinically established for noncardiac nucleic‐acid delivery [25–27].
Liposomes	~50–200 nm	Hydrophilic and hydrophobic small molecules; proteins/peptides	Primarily IV	High	RES uptake, complement activation, phospholipid oxidation, aggregation, and premature cargo leakage	Clinically established as drug carriers, including liposomal DOX; cardiac‐targeted applications remain predominantly preclinical [28].
Solid‐lipid nanoparticles (SLNs)	~100–200 nm	Poorly water‐soluble small molecules; sustained‐release drugs	Oral; IV	High	Limited hydrophilic cargo loading, polymorphic lipid transitions, and drug expulsion during storage	Predominantly preclinical for cardiovascular applications [29, 30].
Polymeric nanoparticles (PNPs; e.g., PLGA)	~30–200 nm	Small molecules, proteins, peptides, miRNA/siRNA, DNA	IV; local/IM	High for biodegradable polymers such as PLGA	Acidic degradation products, cationic‐polymer cytotoxicity, inflammatory responses, and variable degradation/release kinetics	Extensive cardiovascular preclinical evidence; clinically established polymers exist, but cardiac‐targeted formulations remain investigational [31, 32].
Dendrimers	~1–10 nm	Small molecules, nucleic acids, imaging agents, multivalent ligand/drug conjugates	Primarily IV	Low to variable; generation‐ and chemistry‐dependent	Cationic surface‐mediated membrane toxicity, hemolysis, cytotoxicity, and potential tissue accumulation	Early preclinical cardiovascular development [33, 34].
Metal‐based nanoparticles	~20–50 nm	Small molecules, proteins, imaging agents, thrombolytics; theranostic cargo	IV; local administration	Low to variable, depending on composition/coating	Long‐term tissue accumulation, oxidative stress, endothelial injury, thrombogenicity, and metal‐related toxicity	Primarily preclinical for cardiovascular therapy; some iron oxide systems have established diagnostic/imaging precedent [35, 36].
Carbon‐based nanoparticles	~10–50 nm	Nucleic acids, proteins/growth factors, small molecules; tissue‐engineering applications	IV or local, depending on platform	Generally low	Persistence, oxidative stress, endothelial dysfunction, inflammation, and uncertain long‐term clearance	Early preclinical; greater development in biosensing and cardiac tissue engineering than systemic cardiac drug delivery [37].
Hybrid nanoparticles	~50–200 nm	Small molecules, nucleic acids, proteins/peptides, multimodal therapeutic and imaging cargo	Primarily IV; local delivery also feasible	Composition‐dependent	Increased formulation complexity, component‐specific toxicity, difficult characterization, and batch‐to‐batch reproducibility	Preclinical; promising for multifunctional/theranostic cardiovascular delivery but limited clinical maturity [38, 39].

^a^Size range values are representative ranges and vary with formulation, cargo, and functionalization.

### 2.1. Organic NPs

Organic NPs composed primarily of organic molecules such as lipids, polymers, dendrimers, and biomimetic matrices have attracted significant attention in cardiovascular nanomedicine owing to their excellent biocompatibility, biodegradability, and tunable surface chemistry. They encapsulate small molecules, peptides, proteins, and nucleic acids, while permitting precise control over circulation time, release kinetics, and cardiac targeting. Their biodegradability makes them particularly attractive for CVDs requiring repeated administration, although their performance depends strongly on disease‐specific vascular permeability, tissue architecture, and target‐cell accessibility.

#### 2.1.1. LNPs

LNPs are among the most advanced organic nanocarrier systems, particularly for nucleic acid therapeutics. Their self‐assembled structures, comprising ionizable or cationic lipids, phospholipids, cholesterol, and PEG‐lipids, enable efficient encapsulation and intracellular delivery of small‐molecule drugs and nucleic acids [40, 41]. LNPs protect nucleic acids from enzymatic degradation and can promote endosomal escape following cellular uptake.

For cardiovascular applications, however, the major challenge is overcoming the strong hepatic tropism of conventional LNPs and achieving sufficient myocardial and cell‐specific delivery [42]. This limitation is particularly important in chronic HF, where vascular permeability is relatively limited, whereas injured myocardium following MI may provide a transient opportunity for enhanced accumulation. Surface modification and lipid composition optimization can shift biodistribution toward cardiac tissue and potentially enable cardiomyocyte‐directed RNA delivery. Recent advances in cardiac LNP–RNA delivery therefore support this platform’s potential for transient gene expression, gene silencing, and regenerative therapies, although clinically meaningful cardiomyocyte transfection after systemic administration remains a major translational challenge [43].

#### 2.1.2. Liposomes

Liposomes are phospholipid vesicles with one or more bilayers enclosing an aqueous core, making them suitable for encapsulating both hydrophilic and lipophilic agents [44]. They are among the earliest and most extensively characterized organic nanoscale carriers [45]. Liposomes provide controlled, sustained drug release, enhanced drug stability by preventing degradation, and can be surface modified for targeted delivery to inflamed or ischemic myocardium using ligands, antibodies, or peptides [46, 47]. PEGylation (coating with polyethylene glycol) can prolong circulation and minimize opsonization, thereby improving myocardial uptake and reducing immune clearance [48].

In CVD, liposomes are particularly attractive when prolonged circulation or controlled delivery of small‐molecule cardioprotective agents is required. Following acute MI, longer circulation may increase exposure to transiently permeable injured myocardium, whereas ligand‐functionalized liposomes may be more appropriate for chronic HF, where passive accumulation is limited. In DOX therapy, liposomal formulations also show that modifying drug biodistribution can reduce cardiac exposure, although reduced cardiotoxicity should not be interpreted as cardiomyocyte‐specific targeting. Limitations include storage instability, phospholipid oxidation, aggregation, and reticuloendothelial system (RES) uptake [49–52].

#### 2.1.3. Solid‐Lipid Nanoparticles (SLNs)

SLNs are colloidal organic carriers made of biocompatible lipids that remain solid at room and body temperature and are stabilized by surfactants or emulsifiers [53]. They may be triglycerides, waxes, fatty acids, or their derivatives, which are stabilized with an emulsifier or surfactant [54]. They combine several advantages of liposomes and polymeric NPs (PNPs), offering high stability, controlled release, and the ability to load both hydrophilic and lipophilic drugs, while eliminating the need for organic solvents in synthesis [55, 56].

SLNs improve the bioavailability of poorly water‐soluble drugs and exhibit excellent safety and biodegradability [57]. Their sustained‐release properties may be particularly useful for prolonged treatment of post‐MI remodeling or chronic HF, where repeated systemic dosing may be undesirable. However, SLNs possess limited intrinsic cardiac specificity and generally require ligand functionalization for selective myocardial or cellular delivery. Nonetheless, they may expel drugs during polymorphic transitions during storage, necessitating optimization of lipid composition and crystallinity [58, 59].

#### 2.1.4. PNPs

PNPs are nanoscale carriers fabricated from biodegradable polymers such as poly lactic‐co‐glycolic acid (PLGA), polylactic acid (PLA), and polycaprolactone (PCL). Their mechanical stability, tunable degradation, and modifiable surfaces enable sustained delivery of small molecules, peptides, proteins, and nucleic acids [60].

PNPs can be engineered for controlled release profiles, extending therapeutic action over days to weeks, and surface‐functionalized with cardiac‐specific ligands (e.g., CTP, RGD) to enhance myocardial targeting. Polymeric carriers have shown efficacy in reducing oxidative stress, inflammation, and cardiac remodeling in preclinical CVD models [61]. Unlike lipid‐based NPs, PNPs are highly versatile and tunable, offering greater stability that enables long‐term storage and prolonged in vivo release profiles. However, challenges remain in optimizing drug loading, release kinetics, and NP stability, while avoiding acidic degradation by‐products and minimizing immune responses that could hinder clinical translation [62].

#### 2.1.5. Dendrimers

Dendrimers are highly branched, monodisperse macromolecules characterized by a central core, repetitive interior layers, and multiple surface functional groups, providing precise control over size and surface chemistry [63, 64]. Their architecture provides vast loading capacity and the ability to conjugate targeting ligands or therapeutic molecules for cardiac applications. Dendrimers have shown potential for controlled and sustained drug or gene delivery, nitric oxide release, and antioxidative therapies in CVDs [65].

Their advantages include multivalency, tunable surface charge, and intracellular trafficking capacity, which enhance the solubility and bioavailability of cardioprotective agents. However, higher‐generation dendrimers may exhibit cytotoxicity and cardiotoxicity, primarily because excess positive charge disrupts cell membranes [66]. Surface modification with neutral or PEG groups mitigates these effects and improves safety for biomedical use [67].

Overall, organic NPs provide a versatile, biocompatible platform for developing CVD‐specific nanomedicines. By tailoring composition, size, charge, and surface chemistry, each class offers distinct pharmacokinetic and biodistribution advantages suitable for different cardiovascular applications, from cell‐specific targeted small‐molecule delivery to nucleic‐acid therapeutics and gene repair systems.

### 2.2. Inorganic NPs (INPs)

INPs include metal‐, metal oxide‐, and carbon‐based nanomaterials and possess distinctive physical, chemical, and catalytic properties. Their high surface area, structural tunability, magnetic and optical behaviors, and robustness make them attractive platforms for cardiovascular diagnostics and therapeutics. They are employed for simultaneous drug delivery, molecular imaging, and modulation of cellular signaling in CVD. However, despite these benefits, INPs may induce oxidative stress, inflammation, and genotoxicity, underscoring the need for rigorous toxicity assessment and surface engineering before clinical use. The section below discusses important classes of INPs.

#### 2.2.1. Metal‐Based NPs

Metal‐based NPs, including iron oxide, gold, and silver NPs, have been extensively explored for drug delivery, imaging, and modulation of inflammatory pathways in CVDs [68, 69]. Engineered at the nanoscale, they have reproducible physical, chemical, and biological properties, including a large surface area, tunable electrical, magnetic, and optical properties, and amenability to functionalization with a wide range of biomolecules. With typical sizes ranging from 10 to 100 nm, these NPs interact readily with cardiac and vascular cells, enabling targeted deposition in atherosclerotic plaques, ischemic myocardium, and infarct zones [68]. Iron oxide NPs serve as both carriers and magnetic resonance imaging (MRI) contrast agents, facilitating real‐time visualization of cardiac injury and treatment response [69, 70].

Gold NPs exhibit noteworthy chemical stability and optical properties that enable photothermal therapy and redox modulation, reducing oxidative damage in ischemia–reperfusion injury [71]. Silver NPs have potent antimicrobial and anti‐inflammatory properties relevant to stent coatings and post‐surgical cardiac applications [68]. Because of their nanoscale size and tunable surfaces, they can be engineered to enhance interactions with cardiovascular tissues, potentially circumventing the limitations of traditional therapies [72]. Surface functionalization with ligands, peptides, and antibodies improves targeting specificity to injured myocardial or vascular tissue. Superparamagnetic iron oxide NPs are being investigated for CVDs [73, 74]. Transferrin and apolipoproteins attach to their surface to bind receptors such as TfR1 (upregulated after myocardial injury) and LDL receptors, respectively, in the heart, improving delivery to damaged heart tissue [68, 75, 76]. For thrombosis, Fe3O4 NPs have been used to carry tissue plasminogen activator, specifically increasing drug accumulation at the lesion [77].

Nevertheless, long‐term safety concerns persist. If poorly stabilized or uncoated, metal NPs may trigger oxidative stress, inflammation, thrombosis, or DNA damage; thus, biocompatible coatings such as polymers, silica, or biomolecules are essential to improve safety and biodegradability [72, 78].

#### 2.2.2. Carbon‐Based NPs

Carbon‐based NPs, including fullerenes, graphene, and carbon nanotubes (CNTs), exhibit unique electrical conductivity, mechanical strength, and abundant modifiable surface groups, making them useful for drug and gene delivery, biosensing, and cardiac tissue engineering [79, 80]. Fullerenes and graphene oxide have strong antioxidant activity that can mitigate ischemia‐induced oxidative stress [81, 82]. CNTs penetrate cells efficiently and can carry genes, siRNA, or other biomolecules relevant to cardiac regeneration or cardiomyocyte protection [83].

Graphene‐based composites are increasingly incorporated into electrically conductive scaffolds to improve cardiomyocyte coupling, contractile behavior, and tissue repair [84, 85]. Despite their advantages, carbon nanomaterials may cause cytotoxicity, endothelial dysfunction, or promote atherosclerosis if not appropriately functionalized or purified [86]. Long‐term biocompatibility and biodegradation remain critical barriers to their clinical translation [87, 88]. One particular concern is that the persistence of carbon‐based nanomaterials in vivo, coupled with their limited biodegradation pathways, poses challenges for chronic cardiovascular applications that require repeated dosing [89].

### 2.3. Hybrid NPs

Hybrid NPs are second‐generation nanomaterials in which both organic and inorganic components are integrated within a single nanostructure, combining the biocompatibility of organic materials with the magnetic, optical, or catalytic capabilities of inorganic cores [90]. Examples include polymer–metal oxide particles, lipid–magnetic NPs, and polymer–lipid–metal assemblies designed for controlled release, imaging, and tissue repair [91].

Hybrid systems can encapsulate both hydrophilic and hydrophobic drugs, protect labile therapeutic agents, and allow dual therapeutic and diagnostic (theranostic) functionality. Magneto‐liposomes and lipid–polymer hybrid NPs show improved targeting, extended circulation, and sustained release in MI and vascular remodeling models [92]. The hybrid architecture supports the delivery of diverse therapeutic agents, such as nucleic acids, peptides, and growth factors, to injured myocardium, thereby promoting angiogenesis and tissue repair. In other models, lipid‐polymer hybrid NPs with PLGA cores and lipid‐PEG shells show greater stability and controlled release than conventional liposomes [93]. However, synthesis complexity, safety concerns associated with inorganic components, and challenges with large‐scale reproducibility remain significant obstacles. Overcoming these manufacturing and safety challenges is essential for successful clinical translation.

### 2.4. EVs as Emerging Biological Nanocarriers

EVs, including exosomes and other membrane‐derived vesicles, have emerged as biological nanocarriers for cardiovascular drug delivery [94–96]. Unlike conventional synthetic NPs, EVs are naturally derived lipid‐bilayer vesicles that mediate intercellular communication by transporting proteins, lipids, and nucleic acids [97, 98]. Although EVs are not conventional engineered NPs, their nanoscale dimensions, intrinsic biocompatibility, ability to protect biological cargo, and potential for surface engineering make them relevant complementary platforms for cardiovascular nanomedicine [99, 100]. EVs can also be loaded or engineered to deliver therapeutic RNAs, proteins, and small molecules and modified with targeting ligands to improve cardiac or cell‐specific delivery [101, 102].

From the disease‐specific perspective emphasized in this review, EVs may be particularly attractive for MI and cardiac regeneration, where endogenous intercellular signaling, inflammation, and tissue injury can potentially be exploited for therapeutic delivery. Researchers have investigated engineered EVs to enhance myocardial retention and deliver cardioprotective or regenerative cargo to injured cardiac tissue [103]. However, cardiac accumulation does not necessarily translate into cardiomyocyte‐specific uptake, and the relative contribution of EV‐mediated targeting versus intrinsic biological activity remains an important consideration.

Despite their promise, EVs present translational challenges distinct from those of synthetic NPs, including heterogeneity in cellular origin and composition, variable cargo loading, purification and characterization difficulties, batch‐to‐batch variability, limited large‐scale production, and challenges in establishing standardized, GMP‐compatible manufacturing [104]. Therefore, EVs should currently be considered complementary biological nanocarriers rather than direct replacements for well‐defined synthetic NP platforms. Further head‐to‐head studies are needed to determine whether engineered EVs provide meaningful advantages in cardiac targeting, intracellular delivery, safety, and therapeutic efficacy compared with conventional NPs.

Taken together, the therapeutic value of a NP platform in CVD is determined not simply by its material composition but by how effectively its physicochemical properties address the biological barriers of the intended disease. This disease–platform relationship underpins the subsequent discussion of size, surface charge, biocompatibility, targeting strategies, and disease‐specific NP design.

## 3. Properties Relevant to Cardiac Targeting

Effective cardiac‐specific drug delivery depends heavily on NP physicochemical properties. Characteristics such as size, shape, surface charge, and biocompatibility govern biodistribution, circulation time, cellular uptake, and myocardial retention. For biologically derived platforms such as EVs, cellular origin, membrane composition, cargo heterogeneity, and surface engineering also determine biodistribution and targeting. Understanding how these properties influence NP–heart interactions is essential for optimizing nanocarriers for cardiomyocyte and disease‐specific targeting (Figure 1).

**Figure 1:**
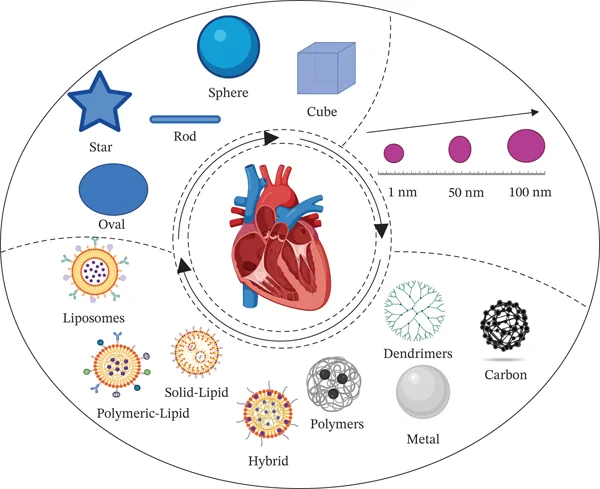
Design parameters governing nanoparticle‐based drug delivery to the heart. Schematic overview of key nanoparticle design features relevant to targeted cardiac drug delivery. The figure highlights major nanoparticle classes, including liposomes, polymeric and solid‐lipid nanoparticles, hybrid systems, dendrimers, and inorganic platforms (metal‐ and carbon‐based), together with representative particle geometries (e.g., spherical, rod‐shaped, cubic, and anisotropic forms) and representative nanoparticle size ranges (1–200 nm). Variations in nanoparticle size, shape, and material composition critically influence circulation time, pharmacokinetics, biodistribution, endothelial transport, myocardial penetration, and cellular or subcellular targeting, ultimately determining therapeutic efficacy and safety in cardiovascular disease models.

These physicochemical properties do not act independently. Instead, they interact with the disease‐specific environment, which can affect NP behavior in the body. For example, NP formulation may affect accumulation in the heart, cellular uptake, and therapeutic efficacy across different heart diseases. In acute MI, increased vascular permeability and intense inflammation may facilitate NP delivery, whereas in chronic HF, fibrotic tissue and decreased blood flow may hinder delivery [105]. Thus, NP size, surface charge, and biocompatibility should be considered based on the cardiac disease they are designed for.

### 3.1. Size and Shape

NP size plays a decisive role in biodistribution, clearance, and myocardial retention. Studies in ischemia–reperfusion models indicate that NPs between 20 and 200 nm exhibit significantly greater passive accumulation in injured myocardium than very small (< 10 nm) or very large particles [106]. Particles below 10 nm are rapidly cleared through renal filtration, whereas particles above 200 nm are preferentially sequestered by the liver and spleen, limiting their cardiac accessibility. Representative experimental cardiac‐targeting systems, such as ligand‐functionalized liposomes, have hydrodynamic diameters of ~140 nm to balance circulation time and tissue penetration [107].

However, the optimal NP size varies across cardiac diseases because each disease’s microenvironment differs. In MI, acute inflammation causes increased permeability of blood vessels, which can lead to the passive accumulation of NPs of about 50–200 nm in the injured heart tissue [108]. However, this increase in vessel permeability is short‐lived, as blood vessels gradually recover within a few days after injury. In chronic HF, capillary density decreases and fibrosis increases, which prevent NPs from penetrating the dense extracellular matrix. In atherosclerosis, NP size influences drug delivery; smaller particles can more easily pass through the dysfunctional endothelium and accumulate within the lipid‐rich core of atherosclerotic plaques [109].

Although most cardiac nanomedicine studies use spherical NPs, particle shape also influences internalization, immune recognition, and tissue interaction. Nonspherical geometries such as rods, disks, or bowl‐shaped particles may exhibit enhanced adhesion to cardiac or endothelial surfaces, altered blood flow dynamics, and improved cellular uptake [110]. Shape engineering and nanopatterned surfaces can increase contact with cardiomyocytes, promote endocytosis, or modulate immune responses. Researchers are increasingly exploring these structural features to fine‐tune NP–tissue interactions and improve cardiomyocyte‐targeting efficiency.

A major limitation of optimizing NP size and shape for cardiac drug delivery is that most studies have been performed in healthy animals or in rodent models of acute heart injury. These models may not accurately represent the aged, fibrotic, or chronically diseased human heart. In addition, the timing of NP accumulation, especially the short period of passive targeting after MI, has not been thoroughly studied in large animal models or in humans [106, 111].

### 3.2. Surface Charge

Surface charge plays a critical role in NP stability, protein‐corona formation, cellular uptake, and clearance by the RES. NPs with a neutral or slightly negative surface charge generally exhibit prolonged circulation time and reduced macrophage uptake, thereby increasing the likelihood of reaching the heart. This feature is particularly relevant in cardiac diseases such as MI, where vascular inflammation and endothelial dysfunction may influence NP distribution. In contrast, highly positively charged NPs often exhibit enhanced cellular interactions and uptake, but are also associated with increased serum protein adsorption, cytotoxicity, and rapid systemic clearance, which can limit effective cardiac delivery. In some settings, cationic NPs have also been associated with membrane disruption and adverse electrophysiological effects in cardiac tissue [112]. Therefore, partial masking through PEGylation may reduce opsonization and prolong biological half‐life but may impair heart cell entry if used excessively [113].

Surface charge becomes even more important in the injured myocardium, where the local microenvironment is altered by acidosis, oxidative stress, and inflammation [114]. In ischemic regions after MI, extracellular pH may drop to approximately 6.5–6.8, and reactive oxygen species (ROS) levels rise [115]. This has led to the development of charge‐responsive NPs that remain neutral or slightly negative in circulation but acquire a mild positive charge in the acidic environment [116, 117]. Such a shift can enhance interaction with negatively charged cell membranes and improve cellular uptake and endosomal escape.

In atherosclerosis, the acidic milieu in atherosclerotic plaques, with the pH in the necrotic core being ~5.5, represents an opportunity to exploit pH‐responsive NPs [118]. These NPs can change their surface charge in response to the acidic environment, improving macrophage uptake and enhancing drug release. Surface charge is disease‐specific and also relevant to other cardiac diseases. Mitochondrial oxidative stress plays a major role in causing cardiac injury in DOX‐induced cardiotoxicity [119]. Positively charged NPs bearing mitochondrial‐targeting molecules, such as triphenylphosphonium (TPP), have been designed to exploit the negative mitochondrial membrane potential and enhance drug delivery to this organelle [120, 121]. However, the positive surface charge that improves mitochondrial targeting may also increase protein adsorption in blood and increase clearance by the RES. Therefore, the surface charge should be well balanced with stealth properties for efficient circulation and targeting. The protein corona, a layer of blood proteins that forms on the surface of the NP after contact with blood, can alter the behavior of the NP. It can mask the original surface charge, change the effective zeta potential, and cause NPs to accumulate in nontarget organs. This effect may be more pronounced in CVDs, as systemic inflammation modulates plasma protein composition and drives disease‐specific protein‐corona formation [122]. Therefore, evaluating NP–protein interactions in biological fluids obtained from patients with conditions such as MI or HF is important, although such studies are still rarely performed. However, this strategy requires careful optimization, as excessive positive charge may still promote premature clearance or toxicity, whereas strongly negative particles may exhibit limited internalization by cardiomyocytes [123]. Therefore, balancing circulation stability with efficient myocardial and intracellular delivery is a key consideration in designing charge‐sensitive nanocarriers for heart disease.

### 3.3. Biocompatibility

Biocompatibility is essential for preventing inflammatory or toxic reactions during cardiovascular administration. Materials such as lipids, biocompatible polymers (e.g., PLGA), and porous silicon have demonstrated good compatibility with cardiac tissue and low cytotoxicity in vivo [124]. NPs must not interfere with cardiac electrophysiology, vascular integrity, or hemodynamic stability. Surface engineering, including PEGylation and ligand conjugation, improves biocompatibility by reducing immunogenicity, promoting selective uptake, and minimizing off‐target interactions [32]. Comprehensive biocompatibility testing should assess effects on cardiac contractility, rhythm, vascular permeability, hematological parameters, and local tissue response at the administration site. Long‐term safety evaluation is particularly important given the chronic nature of many CVDs and the potential for repeated dosing.

The heart is very sensitive to NP toxicity as it beats continuously, has limited regenerative capacity, and relies on mitochondria for energy production. Therefore, NP safety should be carefully examined by studying their effects on cardiac contractility, heart integrity, blood vessels, and blood compatibility. Another important consideration is immunogenicity [125]. Activation of the immune system can exacerbate cardiac remodeling after MI and accelerate progression of atherosclerosis. Repeated NP administration could also induce complement activation‐related pseudoallergy (CARPA) and anti‐PEG antibody generation, reducing treatment efficacy and increasing the risk of adverse reactions [126]. NP surface properties also modulate the risk of coagulation and thrombosis [36]. Metallic and carbon NPs can induce oxidative stress and endothelial dysfunction [36]. Chronic diseases require a long‐term safety evaluation, and biodegradable materials (PLGA, lipids) are preferred over nonbiodegradable systems to reduce the cumulative toxicity [127, 128]. Disease‐specific considerations include avoiding exacerbation of reperfusion injury in MI, arrhythmias in HF, plaque destabilization in atherosclerosis, and increased oxidative burden in DOX cardiotoxicity.

## 4. Targeting Strategies for Cardiomyocytes

Targeted delivery to cardiomyocytes is a central objective in cardiovascular nanomedicine, enabling therapeutic concentrations at the injury site while minimizing systemic exposure. Two major approaches are used to enhance cardiac or cardiomyocyte specificity: passive targeting, which exploits injury‐induced changes in tissue physiology, and active targeting, which uses molecular ligands to direct NPs to defined receptors on cardiac cells (Figure 2). Advances in ligand selection, NP design, and organelle‐specific targeting have significantly expanded the potential of nano delivery systems for treating heart disease. A one‐size‐fits‐all NP design approach is unlikely to succeed across heterogeneous CVDs, given the distinct biological and microenvironmental differences among cardiac pathologies. Each CVD has distinct biological features; thus, NP design should match the specific disease. The strategies discussed here mainly relate to MI, HF, and DOX‐induced cardiotoxicity, where cardiomyocytes are the main target cells, as atherosclerosis is mainly a disease of macrophages and blood vessels. Passive targeting helps NPs reach and remain in the heart. Active targeting is required for cardiomyocytes to take up NPs and for effective intracellular delivery.

**Figure 2:**
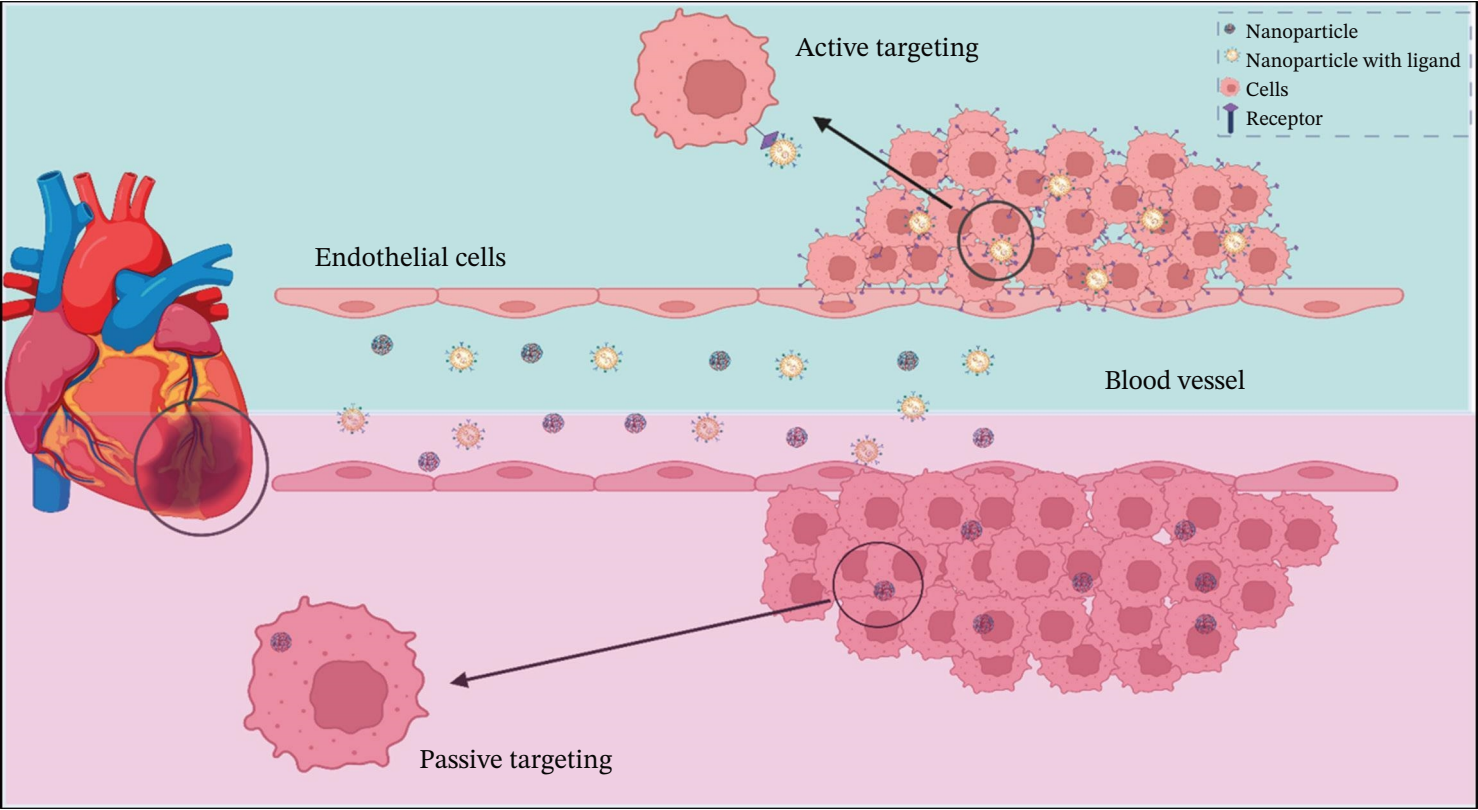
Active and passive targeting mechanisms of nanoparticles to injured tissue. Schematic illustration depicting two major strategies for nanoparticle delivery to injured or diseased tissue. Active targeting involves functionalizing nanoparticle surfaces with specific ligands that recognize and bind receptors overexpressed on activated endothelial cells or injured parenchymal cells. Ligand–receptor interactions facilitate selective binding, cellular uptake, and enhanced intracellular delivery of therapeutic cargo. In contrast, passive targeting in acute cardiac injury relies on transient increases in vascular permeability and endothelial disruption that permit nanoparticle extravasation and retention without ligand‐mediated recognition. Unlike the persistent EPR phenomenon described in tumors, this permeability window in injured myocardium is temporally restricted. Importantly, nanoparticle accumulation within injured tissue does not necessarily indicate cellular internalization or functional intracellular delivery, which require additional targeting and uptake mechanisms.

### 4.1. Passive Targeting

Passive targeting of cardiomyocytes relies on the physicochemical properties of NPs and the altered microenvironment of the diseased myocardium. MI and ischemia–reperfusion injury are associated with increased vascular permeability, inflammatory infiltration, endothelial disruption, and extracellular matrix degradation, all of which promote enhanced NP extravasation and retention. NPs ranging from 20 to 200 nm demonstrate optimal passive accumulation in injured myocardium by traversing leaky vasculature without being rapidly cleared by the kidneys or captured by the RES [106, 129].

The passive targeting in MI is often compared with the enhanced permeability and retention (EPR) effect in tumors. However, there is an important distinction. In tumors, new blood vessels are still forming, so the vessels remain leaky for a long time. In MI, blood vessels leak only briefly after heart injury. Permeability peaks during the first few hours and gradually returns to normal within a few days as inflammation subsides and the vessels heal [130]. Consequently, passive targeting is only successful within this narrow treatment window and not applicable for chronic diseases like HF or stable atherosclerosis.

Ischemia/reperfusion injury, through mPTP opening, is a major cause of mitochondrial dysfunction in cardiomyocytes, leading to cell death. While CsA inhibits mPTP opening, it has various systemic side effects. PLGA NPs loaded with CsA exploit the vascular permeability of the infarcted area to enhance cardiac uptake and minimize systemic exposure [131]. PEGylation and attachment of mitochondria‐targeting peptides, such as SS31, may further optimize this approach by enhancing mitochondrial delivery and reducing oxidative stress, apoptosis, and left ventricular dysfunction. NPs‐mediated cardioprotective delivery promises improved cardiomyocyte survival and functional recovery after IR; however, several challenges persist, including nonspecific uptake and translation to humans. CsA NPs represent passive targeting to mitigate mitochondrial injury in cardiovascular therapy [131].

NPs shape also influences passive uptake. Nonspherical NPs, such as rods or bowl‐shaped structures, exhibit more favorable adhesion and internalization than spherical counterparts, likely due to differences in membrane contact angles and flow dynamics [132, 133]. NP elasticity further affects their ability to cross endothelial barriers and interact with cardiomyocytes. In addition, PEGylation increases circulation time and reduces nonspecific clearance, giving particles more opportunity to reach damaged cardiac tissue [134].

Although passive targeting is advantageous in acute injury settings where vascular permeability is elevated, its effectiveness decreases as healing progresses, limiting long‐term or chronic delivery. Therefore, passive mechanisms are often combined with active targeting strategies to achieve more selective and durable cardiomyocyte engagement. Passive targeting alone is unlikely to be sufficient for chronic cardiac diseases, where vascular permeability is limited and sustained, cell‐specific delivery is required.

The success of passive targeting depends on the disease stage. During the early stage of MI, inflammation and damage cause leaky blood vessels, allowing NPs to enter injured heart tissue more easily. Passive uptake decreases as the heart heals and blood vessels return to normal. With chronic HF, there are fewer blood vessels and more scar tissue, so it is difficult for NPs to passively target the heart tissue [135]. Therefore, passive targeting is usually combined with active targeting approaches to enhance selective delivery to cardiomyocytes. Passive targeting alone is not sufficient for chronic cardiac disease; because vascular permeability is limited and sustained, cell‐specific delivery is required [136].

### 4.2. Active Targeting

Active targeting enhances delivery specificity by functionalizing NPs with ligands that bind to receptors on the cardiomyocyte surface or in the injured myocardium. These ligands include peptides, antibodies, aptamers, and small molecules that recognize overexpressed or disease‐induced surface markers. Receptor‐mediated binding facilitates selective uptake via endocytosis, increases intracellular delivery, and reduces off‐target distribution and systemic toxicity [137]. Active targeting also enables precise delivery of sensitive biological cargo such as siRNA, mRNA, and proteins [138, 139]. In recent years, active strategies have expanded beyond the cell surface to include organelle‐specific targeting, especially mitochondria, lysosomes, nuclei, and the endoplasmic reticulum [140].

Active targeting is particularly relevant for chronic heart diseases, such as HF, cardiomyopathies, and diabetic cardiomyopathy, because passive targeting is ineffective when blood vessels are not leaky. It is preferred to select the targeting molecule (ligand) based on receptors highly expressed in each disease. For example, the angiotensin II type 1 (AT1) receptor is upregulated in the heart after MI and therefore is an attractive target for drug delivery [141]. In HF, the heart attempts to compensate for decreased function by increasing natriuretic peptide receptor (NPR) levels [142]. In DOX‐induced cardiotoxicity, increased mitochondrial oxidative stress provides an opportunity to target drugs to this organelle [143] [144].

Targeting amiodarone delivery with CTP‐functionalized NPs improves cardiac specificity and reduces systemic toxicity [145]. Among the most active Class III antiarrhythmics, amiodarone has serious limitations due to its long half‐life and preferential uptake by noncardiac tissues, such as the liver, lungs, and thyroid [146]. CTP‐conjugated PNPs increase cardiomyocyte uptake two‐ to fivefold, improving antiarrhythmic activity while reducing pulmonary and thyroid toxicity [145]. This is because targeted NP delivery systems, such as this one, provide a precision medication strategy that circumvents most traditional medication limitations to efficiently address the problem of cardiac dysfunction safety [147].

Mitochondria are particularly attractive in CVD because mitochondrial dysfunction contributes to oxidative stress, cell death, and impaired contractility in many heart diseases [148]. NPs modified with mitochondriotropic ligands, such as TPP, or mitochondrial‐targeting peptides accumulate more in the mitochondrial matrix, improving therapeutic efficacy in models of ischemia, oxidative stress, and cardiomyocyte apoptosis [120].

Other organelle‐targeting strategies employ cell‐penetrating peptides, nuclear localization sequences, or lysosomal escape motifs to direct therapeutics to precise intracellular compartments. Multifunctional or hierarchical nanocarriers incorporate several targeting levels‐ tissue, cell, and organelle‐ to traverse multiple barriers and achieve high specificity within the complex architecture of the heart [26, 149].

Stimuli‐responsive nanocarriers represent another emerging approach. These systems release their cargo in response to pH changes, redox gradients, enzyme activity, or oxidative stress, all of which are altered in the ischemic or failing heart. Such responsiveness enhances spatial and temporal control of drug release, reducing systemic toxicity and ensuring on‐demand therapeutic action [148].

Active targeting is particularly important for chronic diseases such as HF, cardiomyopathies, and diabetic cardiomyopathy, where passive mechanisms alone are insufficient due to limited or absent vascular leakage.

### 4.3. Dual‐Targeting and Multilevel Responsive Nanocarriers

Conventional organelle‐targeting strategies such as TPP or SS‐31 address only the final intracellular step of drug delivery. Recent nanocarriers increasingly combine tissue‐, cell‐, and organelle‐level targeting or integrate targeting with disease‐responsive activation to overcome sequential biological barriers. For example, injured‐cardiac‐targeting magnetic nanovesicles developed for IL‐10 mRNA delivery combine injury‐selective nanovesicle targeting with magnetic guidance, producing enhanced accumulation in injured cardiomyocytes and infarcted myocardium and improving post‐MI remodeling [150]. Similarly, macrophage membrane‐coated resveratrol NPs exploit the intrinsic homing of inflammatory macrophages to infarcted myocardium while simultaneously providing immune‐evasive and therapeutic functions, illustrating how biomimetic targeting can integrate circulation, lesion recognition, and local drug delivery [151].

Multilevel designs can further combine cardiac or cell‐specific targeting with intracellular stimuli‐responsive release. In DOX cardiotoxicity, recent multifunctional nanomedicines integrate cardioprotective and anticancer functions by regulating mitochondrial ROS, autophagy, and tumor‐directed drug delivery, demonstrating how multiple biological processes can be addressed within a single platform [152]. Mechanistically, these systems aim to achieve a sequential process: circulation to disease‐site accumulation to target‐cell uptake to intracellular/organelle localization to stimulus‐responsive cargo release rather than relying on a single targeting ligand.

However, increasing nanocarrier complexity can alter particle size, surface charge, protein‐corona formation, pharmacokinetics, immunogenicity, and manufacturing reproducibility. Therefore, to demonstrate the benefit of dual‐ or multilevel targeting, compare cardiac accumulation, target‐cell uptake, intracellular localization, and therapeutic efficacy with corresponding single‐targeted and nontargeted controls.

## 5. Injury‐Induced Ligands and Receptor Targets for Cardiomyocyte‐Specific Delivery

Cardiac injury alters the expression of numerous surface receptors and ligands, creating molecular signatures that can be exploited for precise NP targeting. Among these, NPRs, integrins, and immunologically driven injury markers represent the most validated and biologically relevant classes for cardiomyocyte‐directed delivery.

### 5.1. NPRs

The natriuretic peptide system consists of atrial natriuretic peptide (ANP), brain natriuretic peptide (BNP), and C‐type natriuretic peptide (CNP), along with their receptors GC‐A, GC‐B, and the clearance receptor NPR‐C. ANP and BNP are predominantly expressed in cardiac tissue, whereas CNP is mainly produced by endothelial cells [153, 154]. GC‐A and GC‐B receptors mediate downstream signaling through cyclic guanosine monophosphate (cGMP), producing vasodilatory, antifibrotic, and cardioprotective effects [155]. NPR‐C, although traditionally described as a clearance receptor, also signals through inhibitory G‐proteins to reduce intracellular cAMP levels, thereby contributing to vascular and myocardial protection [156, 157].

NPs functionalized with natriuretic peptide‐mimetic peptides or receptor‐binding motifs can exploit these receptor profiles to achieve targeted adhesion and internalization in cardiomyocytes under stress or HF conditions. This receptor‐driven strategy is beneficial in diseases where natriuretic peptide levels and receptor expression are elevated as part of compensatory remodeling. Whereas the therapeutic utility of NPR targeting must be carefully balanced against the physiological role of natriuretic peptides, as excessive receptor engagement could affect hemodynamic homeostasis. Further, BNP levels vary substantially with disease severity and are influenced by renal function and age, specific variability in targeting efficacy [158].

### 5.2. Integrins

Integrins are transmembrane receptors essential for mechano‐transduction and cell‐extracellular matrix communication. Cardiac injury and remodeling lead to dynamic changes in integrin expression, particularly of the *α*7*β*1D isoform, which is enriched in mature cardiomyocytes [159]. Integrin dysregulation may lead to arrhythmias and contractile dysfunction, which also would indicate the necessity for selective targeting [160] [161]. Upregulation of *α*7*β*1D during ischemia or pressure overload increases cell survival by stabilizing calcium homeostasis and reducing apoptosis [162]. Because integrins play central roles in structural remodeling, peptides such as RGD (Arginine‐Glycine‐Aspartic acid), antibodies, or small‐molecule ligands targeting integrin isoforms have been conjugated to NPs to enhance selective delivery to injured myocardium [163]. This approach improves NP retention, uptake, and therapeutic efficacy by directing nanocarriers toward areas undergoing active mechanical and structural remodeling.

Integrin targeting is useful during post‐MI remodeling, as integrin expression is upregulated in healing myocardium [164]. However, integrins are also expressed on endothelial cells, fibroblasts, and immune cells, reducing cardiomyocyte specificity. RGD‐functionalized NPs may bind activated endothelium and inflammatory cells, so targeting specificity requires careful interpretation.

### 5.3. Autoantigens and Immune‐Related Targets

Cardiac injury exposes intracellular proteins and neoepitopes on the surface of apoptotic or necrotic cardiomyocytes, forming new targets for delivery systems. For example, bispecific antibodies (BiAbs), designed to recognize both therapeutic cells (e.g., stem cells expressing CD45) and cardiac injury antigens such as myosin light chain, have been shown to significantly increase cellular homing and retention in infarcted tissue [165]. Furthermore, in autoimmune or immune‐mediated cardiomyopathies, pathogenic autoantibodies against *β*‐adrenergic receptors or other cardiac proteins contribute to ongoing tissue damage. Targeted removal or neutralization of these antibodies using NP platforms may prevent further cardiomyocyte loss and promote functional recovery [166]. Using autoantigens as targeting moieties raises important safety considerations, as these epitopes are expressed only on injured or dying cells. Off‐target binding to healthy cardiomyocytes is unlikely, but NP accumulation could exacerbate the inflammatory response to dying cells [167, 168].

### 5.4. Damage‐Associated Molecular Patterns (DAMPs)

Ischemia, reperfusion injury, and oxidative stress expose DAMPs on cardiomyocytes, which act as natural ligands for circulating immune factors. Natural IgM antibodies rapidly recognize these DAMPs and can trigger complement activation, exacerbating post‐ischemic injury [169]. NPs designed to bind the same DAMP structures, or to block IgM–DAMP interactions, may reduce inflammatory damage and serve as a targeted therapeutic entry point into injured myocardium. Researchers are currently developing regulations of such innate antibody activity as a novel therapeutic strategy to dampen inflammation and limit myocardial damage [170]. The above targeted approaches have complementary mechanisms to protect and restore injured cardiac tissue [169].

Table 2 summarizes a comparative overview of passive and active targeting strategies across major CVDs.

**Table 2:** Comparison of passive and active nanoparticle targeting strategies in cardiovascular diseases.

Disease	Passive targeting	Active targeting	Targets	Advantages	Limitations	References
Myocardial infarction	Leverages temporary vascular leakiness and inflammation at sites of infarcted myocardium.	Nanoparticles modified with ligands that target injured cardiomyocytes or inflammatory endothelial cells	VCAM‐1, ICAM‐1, integrins, necrosis markers	Capable of concentrating on areas of ischemia; targeted drug delivery offers better efficacy and selectivity	Transient and heterogeneous vascular permeability; limited therapeutic window and rapid systemic clearance.	[91, 171, 172]
Atherosclerosis	Inflamed plaque accumulation through dysfunctional endothelium and increased permeability	Targeted therapy aimed at macrophages, foam cells, and endothelial cells through specific ligands	Macrophage scavenger receptors (SR‐A, CD36), VCAM‐1	High selectivity for immune cells present in plaque; increased treatment specificity	Plaque heterogeneity; uptake in other organs such as liver and spleen	[173–175]
Heart failure	Restricted passive uptake because of decreased perfusion and limited vascular permeability	Cell targeting through peptides/antibodies to specific cardiomyocytes, fibroblasts, or neurohormonal signaling pathway receptors	*β*‐adrenergic receptors, angiotensin receptors, fibroblast‐specific proteins	Permits selective cell targeting despite diffuse disease conditions	Poor nanoparticle penetration; receptor variability	[171, 176]
Cardiotoxicity	Passive accumulation is generally limited and depends on disease‐associated changes in vascular permeability and low‐grade inflammation	Targeting oxidative stress markers, apoptotic cells, or mitochondrial pathways	ROS‐sensitive systems, mitochondrial targeting ligands	Proactive treatment and cardiomyocyte defense	Pathological changes limit passive accumulation	[177, 178]

## 6. Disease‐Specific NP Design and Applications

NP‐based therapies have been evaluated in a wide range of preclinical models of MI, HF, and chemotherapy‐induced cardiotoxicity, with several platforms advancing toward early translational or clinical investigation. Overall, these studies demonstrate that rationally designed nanocarriers can improve myocardial drug retention, reduce infarct size, attenuate remodeling, and mitigate cardiotoxicity. However, most data come from small‐animal models with short‐term follow‐up and heterogeneous endpoints, and few systems have advanced to late‐stage clinical trials.

Importantly, a nanocarrier’s effectiveness is strongly influenced by the pathological microenvironment of the disease being treated. Acute vascular permeability, acidosis, oxidative stress, inflammation, fibrosis, impaired perfusion, and the identity of the predominant target cell differ substantially among CVDs. These disease‐specific characteristics should therefore guide NP size, surface properties, targeting strategy, and release mechanism rather than applying a uniform NP design across different cardiac conditions. This section summarizes significant preclinical findings, evaluates their robustness, and highlights key translational gaps (Figure 3) [170, 179–181].

**Figure 3:**
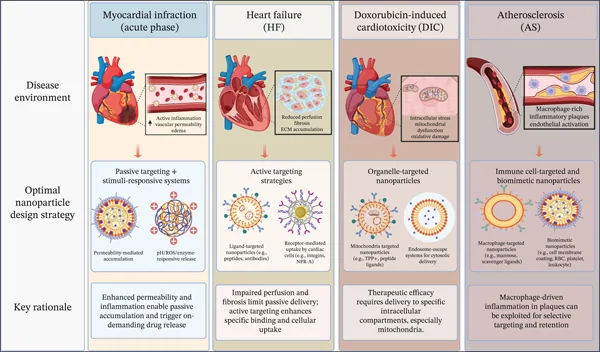
Disease‐specific framework for targeted cardiovascular nanomedicine. Schematic illustrating how disease‐specific cardiac microenvironments dictate optimal nanoparticle design strategies. In myocardial infarction (MI), acute inflammation and increased vascular permeability favor passive targeting and stimuli‐responsive systems. In heart failure (HF), reduced perfusion and fibrosis limit passive accumulation, necessitating active targeting strategies. In doxorubicin‐induced cardiotoxicity (DIC), intracellular and mitochondrial stress signaling requires organelle‐specific targeting. In atherosclerosis (AS), macrophage‐rich inflammatory plaques favor immune cell–targeted and biomimetic nanoparticles. This framework highlights that successful nanoparticle design must align with disease‐specific biological conditions rather than relying on a universal delivery strategy.

### 6.1. MI

A major challenge in MI therapy is the rapid onset of cardiomyocyte death and adverse ventricular remodeling that follow ischemia–reperfusion. At the same time, the infarcted myocardium develops several potentially exploitable features for nano delivery, including transient endothelial disruption and increased vascular permeability, extracellular acidosis, excessive ROS, and recruitment of neutrophils and monocytes/macrophages. Early infiltration of pro‐inflammatory macrophages amplifies tissue injury, whereas subsequent reparative macrophages contribute to angiogenesis and scar formation [170, 179, 180, 182]. Thus, MI may be particularly suitable for NPs designed to passively accumulate during the acute permeability window or for pH‐, ROS‐, and inflammation‐responsive targeting and release [183]. Importantly, these features evolve rapidly after injury, making the timing of NP administration a critical consideration.

Several NP platforms have been designed to deliver cardioprotective, pro‐angiogenic, or regenerative agents directly to ischemic myocardium. Hydrogen sulfide–releasing NPs (HA@ZnS NPs), generated via biomimetic mineralization, exemplify a targeted strategy in which drug release is triggered by the acidic microenvironment of ischemic tissue [184]. In rodent models, these particles improve left ventricular ejection fraction and fractional shortening, reduce end‐systolic volume, limit infarct size, and promote angiogenesis and anti‐apoptotic signaling. This example illustrates how a disease‐associated physicochemical feature, ischemic acidosis, can be incorporated directly into NP design to achieve spatially controlled therapeutic release.

Similarly, growth factor–loaded NPs and squalene‐based gold nanocarriers have been used to provide sustained cardio protection and preserve cardiomyocyte architecture [185]. Other approaches use composite or multifunctional systems, such as conductive hydrogels containing lignosulfonate‐doped polyaniline nanorods and adeno‐associated virus encoding VEGF, which activate pro‐angiogenic pathways including VEGFR2 and PI3K, thereby enhancing vascular regeneration [186].

Glutathione‐responsive NPs have been designed to release therapeutics selectively to activated fibroblasts in the infarct zone, and mitochondria‐targeted lipid–polymer nanocarriers loaded with calycosin and tanshinone show high cardiac accumulation and decreased infarct size in experimental models [61, 187, 188]. These studies also demonstrate that “cardiac targeting” after MI can involve different target cells or compartments, including cardiomyocytes, fibroblasts, inflammatory cells, endothelial cells, and mitochondria, and should therefore not automatically be interpreted as cardiomyocyte‐specific delivery.

Reformulation of natural compounds such as curcumin into polymeric nanocarriers has yielded “nanocurcumin” systems that reduce oxidative stress and inflammatory cytokines while preserving cardiac function. Nanocurcumin formulations, particularly polymeric micelles, reduce oxidative stress, inflammatory cytokines, and myocardial injury markers [189]. These carriers are highly biocompatible and enable targeted delivery, ultimately preserving heart function and limiting infarct expansion [189]. In contrast, cationic gold NPs loaded with transcription factors such as Gata4, Mef2c, and Tbx5 have successfully reprogrammed fibroblasts into cardiomyocytes, reducing scar tissue and improving function post‐MI [190]. Similarly, PLGA‐PEI nanocarriers that deliver 5 ^′^Azacytidine and miR‐133a induce fibroblast‐to‐cardiomyocyte conversion, suggesting a powerful regenerative approach post MI [191].

Gene therapy to improve cardiac function post‐MI has also benefited from NP delivery platforms. For example, reducible dendrimer polymers carrying relaxin‐expressing plasmid DNA target infarct sites, significantly enhancing left ventricular systolic function and modulating extracellular matrix remodeling [192] [193]. LNPs delivering lncRNA‐TARID have shown therapeutic benefits in both murine and porcine models of myocardial fibrosis [194]. The inclusion of a porcine model is particularly important because large‐animal validation remains uncommon in cardiovascular nanomedicine and provides greater translational relevance than rodent efficacy alone.

Beyond preclinical success, several NP systems are approaching clinical translation. Core–shell poly (N‐isopropylacrylamide) nanogels encapsulating tissue plasminogen activator and Y‐27632 have demonstrated rapid fibrin degradation and improved ventricular function following intracoronary administration in experimental models [61].

Taken together, these studies strongly support the concept that NPs can enhance local drug concentration, provide controlled release, and engage disease‐relevant targets such as fibroblasts, mitochondria, and endothelial cells. However, critical limitations remain: most experiments use young, healthy rodents subjected to acute MI, with short follow‐up and relatively high NP doses. Large‐animal data are sparse, and very few studies directly compare NP formulations against optimized free‐drug regimens or established cardioprotective strategies. Moreover, reductions in infarct size and improvements in ejection fraction are often modest and may not translate directly into long‐term clinical benefit.

An additional limitation is that the delivery advantage created by acute MI is transient: vascular permeability, inflammatory‐cell composition, ROS production, and tissue pH change substantially from the acute inflammatory phase to the reparative and fibrotic phases. A NP optimized for delivery within hours of reperfusion may therefore perform differently days or weeks after MI. Therefore, the durability of benefit beyond weeks, the impact on arrhythmia risk, and the influence of comorbid conditions such as diabetes, hypertension, and aging on NP biodistribution and efficacy remain unanswered. Additionally, head‐to‐head comparisons of different targeting strategies (e.g., fibroblast vs. cardiomyocyte vs. endothelial targeting) and delivery routes (intravenous, intracoronary, intramyocardial) are also lacking.

### 6.2. HF

HF is one of the leading causes of death worldwide. Unlike MI, HF is a chronic inflammatory and fibrotic state characterized by persistent macrophage activation, fibroblast expansion, extracellular matrix remodeling, and sustained cytokine signaling [195]. These conditions lack the pronounced vascular leakage observed in acute MI, thereby limiting passive NP accumulation. These features favor prolonged‐circulating and actively targeted systems and make particle size and tissue penetration particularly important design considerations. In chronic HF, NP‐based therapies aim to attenuate maladaptive remodeling, improve contractility, and modulate cell–cell or cell–matrix signaling. Because HF involves multiple interacting cell populations, the desired therapeutic mechanism should determine whether cardiomyocytes, activated fibroblasts, endothelial cells, or inflammatory cells represent the appropriate NP target.

A notable example is the inhalable CTP‐modified calcium phosphate NP for TP‐10 delivery [196]. In preclinical models of pressure overload–induced HF, this system shows selective accumulation in cardiomyocytes and fibroblasts, an acceptable safety profile with repeated inhalation, and improved cardiac function. Mechanistically, TP‐10 modulates cAMP/AMPK signaling in cardiomyocytes and cGMP/PKG pathways in fibroblasts, suggesting coordinated effects on contractility and fibrosis [196]. This study is particularly relevant to chronic HF because it combines active cardiac targeting with a noninvasive route potentially suitable for repeated administration, rather than relying on injury‐induced vascular leakage.

The inhalation route is an emerging strategy for cardiac nanomedicine because pulmonary administration offers a noninvasive approach for repeated dosing while potentially facilitating NP access to the systemic circulation and heart. This may be particularly advantageous for chronic conditions such as HF, where repeated intravenous administration is less desirable. However, pulmonary deposition, mucociliary clearance, alveolar uptake, particle size‐dependent lung retention, and potential pulmonary toxicity can strongly influence systemic and cardiac bioavailability. Therefore, the apparent cardiac benefit of inhaled NPs should be distinguished from the effects of the targeting ligand itself, and direct comparisons among inhaled targeted NPs, nontargeted NPs, and intravenous administration are needed to establish the specific advantage of this delivery route.

However, the available evidence derives from a pressure‐overload model in male mice, limiting conclusions regarding sex differences, HF phenotype, and translation to advanced human HF. Although repeated inhalation showed an acceptable pulmonary safety profile under the reported experimental conditions, longer‐term effects on lung function and the durability of cardiac benefit remain to be established. Moreover, direct comparison with equivalent free TP‐10 and intravenous NP delivery is needed to define the inhalation route’s specific advantage.

Peptide‐guided NPs, by contrast, have more broadly shown improved myocardial uptake, prolonged drug exposure, and reduced systemic toxicity [197]. Platforms incorporating cardiomyocyte‐selective ligands or heart‐targeting peptides have delivered small molecules, RNA therapeutics, and antifibrotic agents, yielding positive functional outcomes in rodent models.

Despite promising results, several issues complicate interpretation. Many HF studies employ early or mid‐stage models rather than advanced, decompensated disease and often lack direct comparison with guideline‐directed medical therapy. Furthermore, many studies leave the relative contribution of improved targeting versus simple pharmacokinetic improvements (e.g., longer half‐life) unclear. HF itself is also heterogeneous; HFrEF, HFpEF, ischemic HF, pressure‐overload HF, and genetic cardiomyopathies differ in perfusion, fibrosis, inflammation, and cellular pathology. Consequently, a delivery platform validated in one HF model should not automatically be assumed to perform similarly across HF phenotypes.

The reproducibility of inhalation‐based and peptide‐guided targeting strategies in large animals and across HF etiologies (ischemic, hypertensive, genetic) remains largely untested. In HF settings, whether NP systems can meaningfully alter hard clinical endpoints (hospitalization, survival), how they interact with neurohormonal blockades, and whether chronic administration will induce immunogenicity, anti‐peptide antibodies, or NP accumulation in off‐target tissues require clear direction and investigation.

The pathophysiology of HF presents unique challenges and opportunities. Physical barriers imposed by the fibrotic microenvironment with dense ECM and activated fibroblasts favor smaller NPs (< 100 nm) with active targeting. Macrophages define the ongoing inflammatory condition, providing targets for immunomodulatory NPs. The chronicity of HF allows sustained release, unlike the transient EPR effect in MI, but it still requires active targeting to overcome perfusion deficits [176].

Given the central role of macrophages in fibrosis and chronic inflammation, NP‐mediated modulation of macrophage polarization (M1 to M2 transition) has emerged as a promising therapeutic strategy to attenuate adverse remodeling and improve cardiac function [170, 179, 198].

NP approaches should be framed within the HF therapeutic landscape. Standard‐of‐care therapies such as ACE inhibitors, beta blockers, mineralocorticoid receptor antagonists, and SGLT2 inhibitors reduce hospitalization and mortality. Karakasis et al. reviewed mechanisms by which SGLT2 inhibitors reduce inflammation, fibrosis, and oxidative stress, and improve mitochondrial function, autophagy, and TGF‐*β*/Smad, STAT3, and mTOR signaling [199]. NP strategies will have to show added benefits compared with these therapies—improved efficacy, reduced side effects, or targeting of mechanisms not covered by existing drugs.

### 6.3. Atherosclerosis

Atherosclerosis is included here as a contrasting CVD in which NP targeting is directed predominantly toward vascular and immune compartments rather than cardiomyocytes [200]. Atherosclerosis is a chronic inflammatory vascular disease characterized by endothelial dysfunction, retention and oxidation of low‐density lipoproteins, recruitment of circulating monocytes, and formation of macrophage‐derived foam cells within the arterial wall [200, 201]. As plaques progress, the lesion microenvironment becomes enriched in inflammatory cytokines, ROS, proteolytic enzymes, apoptotic debris, and, in advanced lesions, necrotic cores and thrombogenic material [202]. Atherosclerosis represents a fundamentally different nano‐delivery environment because the principal therapeutic targets are located within the diseased vasculature. Therefore, NPs designed for atherosclerosis should primarily exploit endothelial, immune‐cell, lipid/plaque, or thrombotic targets. Nanomedicine for atherosclerosis targets macrophages, dendritic cells, foam cells, activated endothelium, extracellular matrix, and thrombotic plaque compounds [200]. This is fundamentally distinct, as the microenvironment and NP design requirements differ substantially from cardiomyocyte‐directed strategies [173, 203, 204].

In this setting, endothelial activation, increased adhesion‐molecule expression, macrophage accumulation, and foam cell formation provide important biological cues for selective delivery. NPs can therefore be engineered to target macrophages, endothelial cells, or plaque components, while ROS‐responsive and biomimetic systems can exploit the oxidative and inflammatory milieu to enhance local payload release. This strategy is particularly attractive because macrophages are central to plaque progression, instability, and eventual rupture. Macrophage‐targeted nanomedicine is a major theme in current atherosclerosis work and has been proposed for both treatment and imaging of unstable plaques. The plaque microenvironment poses unique delivery challenges. Variations in lipid core, fibrous cap thickness, calcification, and inflammation create differential NP accessibility. Thick fibrous caps limit penetration, whereas vulnerable plaques with thin caps and high inflammation are more accessible. This heterogeneity complicates one‐size‐fits‐all strategies and suggests plaque phenotyping for optimal patient selection [205].

These disease characteristics also impose important design constraints. NPs must circulate sufficiently long to interact with diseased vascular regions while minimizing competing uptake by hepatic and splenic macrophages. Particle size, charge, protein‐corona formation, and ligand density therefore strongly influence the balance between plaque localization and reticuloendothelial sequestration. Iron oxide and other imaging‐capable NPs provide an additional opportunity for theranostic approaches in which plaque inflammation or thrombosis can be visualized while simultaneously delivering therapy.

Several NP platforms in atherosclerosis, including liposomes, PNPs, HDL‐mimetic NPs, magnetic NPs, and macrophage membrane‐coated biomimetic systems, with applications ranging from anti‐inflammatory drug delivery to plaque imaging and theranostics, have been explored [206]. These systems have been used to reduce vascular inflammation, improve cholesterol efflux, modulate macrophage phenotype, stabilize vulnerable plaques, and, in some cases, provide simultaneous diagnostic imaging and therapeutic intervention. Macrophage‐targeted and multifunctional NPs have shown promise in reducing plaque burden and promoting plaque stabilization in preclinical studies [207]. Recent reviews emphasize that multifunctional and macrophage‐focused NPs can suppress pro‐atherogenic macrophage functions and improve plaque stability [208].

Atherosclerosis strongly illustrates how immune‐cell biology can guide NP selection. Because macrophages and foam cells are abundant within plaques, macrophage‐targeted delivery may be more beneficial than nonspecific vascular accumulation alone. Likewise, ROS‐responsive NPs may be particularly effective in oxidative plaque environments, whereas ligand‐directed or biomimetic particles may improve binding to activated endothelium and inflammatory cells. A one‐size‐fits‐all NP design approach is unlikely to succeed across heterogeneous CVDs, particularly in atherosclerosis, where immune cell–driven pathology predominates. Contemporary vascular nanomedicine literature consistently highlights this disease‐tailored approach.

Several target‐accessibility issues remain in atherosclerosis nanomedicine. First, the vascular endothelium acts as a barrier that must be penetrated to deliver drugs into plaque macrophages. Passive penetration through fenestrated endothelium covering plaques, along with targeted interaction with adhesion molecules VCAM‐1 and ICAM‐1, increases endothelial penetration. In addition, after entering the plaque, NPs must pass through the dense extracellular matrix and interact with lipid‐rich macrophages [209]. However, plaque biology is highly heterogeneous across disease stage, vascular bed, and individual patients. Moreover, effective contemporary lipid‐lowering and anti‐inflammatory therapies create a high benchmark for demonstrating additional therapeutic benefit. Future studies should therefore quantify plaque‐specific versus liver/spleen biodistribution, evaluate advanced and unstable lesions rather than early plaque formation alone, and determine whether NP treatment improves plaque stability and clinically meaningful outcomes rather than simply reducing lesion area.

### 6.4. DOX‐Induced Cardiotoxicity

DOX is one of the first‐line chemotherapeutics in various malignancies, and cardiotoxicity remains a significant limitation of DOX chemotherapy. In contrast to acute MI, DOX cardiotoxicity does not provide a reliable vascular‐leakage window for passive myocardial targeting. Instead, its disease environment is dominated by cardiomyocyte oxidative stress, mitochondrial dysfunction, iron dysregulation, DNA damage, inflammatory signaling, and progressive cellular injury [210–212]. Therefore, an optimal nanocarrier may need to achieve sequential targeting—first to the heart, then to cardiomyocytes, and, for selected therapeutics, to intracellular organelles such as mitochondria. ROS‐responsive release may offer an additional strategy to exploit the oxidative environment produced by DOX.

Dexrazoxane (DZ), the only FDA‐approved cardio protectant, provides only partial benefit because of its poor cardiac biodistribution, limited intracellular penetration, and instability at physiological pH [213]. In addition, DZ shows potential off‐target effects, including impaired DOX anticancer activity and severe adverse outcomes like myelosuppression and secondary malignancies [214]. These limitations underscore the need for a delivery strategy that specifically enhances DZ accumulation in the heart without compromising DOX’s antitumor efficacy. Therefore, NP strategies have strong potential to deliver cardioprotective agents that selectively shield the heart without interfering with tumor targeting.

Importantly, this therapeutic objective differs from NP formulations designed to deliver DOX to tumors while merely reducing cardiac exposure. The former requires true cardiomyocyte targeting of a cardioprotective cargo, whereas the latter represents pharmacokinetic redistribution of the anticancer agent. This distinction should be maintained when interpreting “cardiac‐specific” NP efficacy.

DOX‐induced cardiotoxicity is characterized by mitochondrial dysfunction, oxidative stress, DNA damage, and sterile inflammation, with activation of innate immune pathways and release of DAMPs [215]. These observations highlight that effective NP strategies in this setting must prioritize intracellular and organelle‐specific targeting rather than relying solely on tissue‐level accumulation. Unlike MI, this condition lacks strong vascular permeability but involves intracellular stress signaling and immune activation, making intracellular and organelle‐specific delivery critical.

This strategy has been explored using multi‐drug co‐assembled nanocomposites incorporating ginsenoside Rb1, probucol, and PI3K*γ* inhibitors, modified with cardiomyocyte‐targeting PCM peptides, which exemplify the cardioprotective strategy [216]. These nanocomposites show selective cardiac accumulation, attenuate oxidative stress and inflammation, promote protective autophagy, and substantially reduce DOX‐induced structural and functional damage while preserving antitumor efficacy [216]. Using a cardiomyocyte‐targeting peptide is particularly relevant here because passive myocardial accumulation alone is unlikely to ensure adequate intracellular delivery to cardiomyocytes.

Further, iron–DOX hyaluronan nanogels coordinate DOX with ferric ions within a hyaluronic acid matrix to achieve CD44‐targeted, lysosome‐localized, pH‐responsive release [217]. These systems lower cardiotoxicity and maintain robust antitumor effects, suggesting that careful control of intracellular release can decouple therapeutic efficacy from cardiac injury. Moreover, Pectin‐based DOX encapsulation has likewise been reported to reduce myocardial lesions and increase Bcl‐2 expression compared with free DOX, indicating a more favorable cardioprotective profile [218]. NP systems that regulate oxidative stress and inflammatory signaling pathways, including macrophage activation and cytokine production, can significantly attenuate DOX‐induced cardiotoxicity, highlighting inflammation as a key therapeutic target [216]. Wang et al. created cerium oxide‐doped Prussian blue NPs (PB@CeO2 NPs) taking advantage of inflammation‐mediated immune cell retention in the injured myocardium [15]. These hybrid nanozymes alleviate mitochondrial damage, inhibit apoptosis, and induce macrophage M2 polarization, while offering MRI contrast for theranostic application. This approach combines the antioxidant and anti‐inflammatory properties with targeted accumulation in inflamed cardiac tissue and is promising for anthracycline‐induced cardiotoxicity [15].

Although these findings are encouraging, most data are derived from short‐term rodent models where tumor burden, DOX exposure, and NP dosing are tightly controlled. The long‐term consequences of chronic NP exposure, particularly in patients with pre‐existing cardiovascular risk factors or those receiving combination chemotherapy, remain unknown. Additionally, potential interactions between NP formulations and the tumor microenvironment (e.g., altered penetration, efflux, or immune modulation) are incompletely characterized. A particularly important translational requirement is to evaluate cardioprotection and anticancer efficacy simultaneously in tumor‐bearing models using clinically relevant cumulative DOX exposure. Studies in healthy animals alone cannot establish whether a cardioprotective NP alters tumor drug delivery or anticancer efficacy. A key debate is whether cardioprotective NP strategies can truly match the robustness and simplicity of existing interventions such as dose limitation, liposomal DOX, or DZ, especially when cost and manufacturing complexity are considered. Comparative studies addressing these questions are currently lacking and need a rigorous approach to translating to the clinic.

### 6.5. Cardiac Transplantation

Cardiac transplantation represents another distinct disease environment because the major therapeutic challenge is immune‐mediated graft injury rather than cardiomyocyte delivery alone. In this context, the cardiac microenvironment is dominated by adaptive immune responses, including T‐cell activation, dendritic cell antigen presentation, and macrophage‐mediated inflammation [219].

The transplantation microenvironment differs fundamentally from ischemic or failing hearts. The transplanted heart faces ongoing allorecognition, where recipient dendritic cells and macrophages present donor antigens to T cells. This immune‐dominant environment requires NP strategies that target immune cells rather than cardiomyocytes. Key approaches include lymph node homing, T‐cell modulation, and regulatory T cell (Treg) induction. These processes create opportunities to target activated immune cells, inflamed graft endothelium, or the transplanted tissue itself. Macrophages also contribute to graft rejection through antigen presentation, cytokine production, and immune cell activation [220]. Activated T cells augment the immune reaction, which can cause graft dysfunction [219]. NP‐based strategies enable targeted delivery of immunosuppressive agents to immune cells and graft tissue, reducing systemic toxicity while improving therapeutic precision [219].

Biomimetic NPs evade immune recognition and accumulate in inflamed graft regions. They carry therapeutic agents to inhibit immune reactions and induce tolerance [22, 198, 219]. Cell membrane–coated NPs (e.g., platelet or mesenchymal stem cell membranes) use natural immune‐evasive properties to prolong circulation, target active inflammation, and reduce off‐target immunosuppression [221]. Tolerogenic NPs induce regulatory T cells or promote anergy in alloreactive T cells, offering long‐term graft acceptance without chronic immunosuppression [222].

Critical translational challenges include the complexity of the alloimmune response across multiple cell types and pathways; the need for long‐term immunosuppression without chronic toxicity; and the difficulty of weaning patients off systemic immunosuppressants. Additionally, patient heterogeneity, varying HLA mismatch, and differences in sensitization status complicate clinical trial design and endpoint selection [223]. The major translational question is whether localized NP‐mediated immunomodulation can reduce systemic calcineurin inhibitor, corticosteroid, or other immunosuppressive exposure while maintaining effective protection against acute and chronic rejection. Long‐term safety is particularly important because transplant recipients require lifelong treatment, and NP accumulation, immune perturbation, infection risk, and malignancy risk must therefore be evaluated over substantially longer periods than in acute MI studies.

Together, these disease‐specific examples demonstrate that cardiovascular nanomedicine should not rely on a one‐size‐fits‐all targeting strategy. Acute MI provides transient vascular permeability, acidosis, ROS, and inflammatory‐cell recruitment that can be exploited for responsive delivery; chronic HF requires strategies capable of overcoming impaired perfusion and fibrotic barriers; atherosclerosis favors vascular and macrophage‐directed targeting; DOX cardiotoxicity places greater emphasis on cardiomyocyte and intracellular/organelle‐specific delivery; and cardiac transplantation provides opportunities for immune‐cell and graft‐directed immunomodulation. Thus, the pathological microenvironment should guide the selection of NP size, surface properties, targeting ligand, cargo, and release mechanism for each cardiovascular indication.

Representative preclinical studies across these CVD settings are summarized in Table 3, highlighting differences in NP platform, therapeutic cargo, targeting strategy, administration route, experimental model, therapeutic endpoint, follow‐up duration, and translational maturity.

**Table 3:** Summary of preclinical nanoparticle‐based therapeutic strategies for cardiovascular diseases.

Disease	Nanoparticle platform	Therapeutic cargo	Targeting ligand/strategy	Route	Model/species	Major endpoint	Follow‐up	Translational maturity	References
Myocardial infarction	H2S‐releasing HA@ZnS NPs	H2S	Acidic microenvironment‐responsive release	IV	Rodent MI	Infarct size, LVEF, angiogenesis	Not clearly specified	Preclinical	[184]
Doxorubicin‐induced toxicity	Cardiac targeting peptides‐modified liposomes	Small molecule	CRPPR (peptide) binds to the cardiac endothelium	IV	Rodent	Cardiomyocyte apoptosis	Not clearly specified	Preclinical	[224, 225]
Heart failure	CTP‐modified calcium phosphate NPs (TP‐10@CaP‐CTP)	TP10 inhibitor	Cardiac‐targeting peptide (CTP; CSTSMLKAC)	Inhalation	Rodent	Cardiac function, cardiac remodeling	6 weeks	Preclinical	[196]
Cardiac transplantation	ROS‐responsive fusion extracellular nanovesicles (FNVs@RAPA)	Small molecule (rapamycin, immunosuppressant)	ROS responsive release	IV	Rodent	Ischemia–reperfusion injury, acute rejection, graft survival	Not clearly specified	Preclinical	[226]
Atherosclerosis	Biomimetic responsive‐targeting hybrid NPs	Small molecules (Atorvastatin)	ROS responsive release	IV	Rodent	Inflammation, lipid metabolism	Not clearly specified	Preclinical	[227]

## 7. Key Considerations in Selecting NP Platforms for Cardiomyocyte‐Targeted Drug Delivery

In CVD, designing an optimal NP system for cardiomyocyte‐specific delivery requires careful consideration of physicochemical properties, biological barriers, drug characteristics, targeting needs, route of administration, safety, and regulatory feasibility, as summarized in Figure 4. Below, we summarize key principles, critically evaluate supporting evidence, and highlight remaining challenges needed to advance NP platforms toward clinical application.

**Figure 4:**
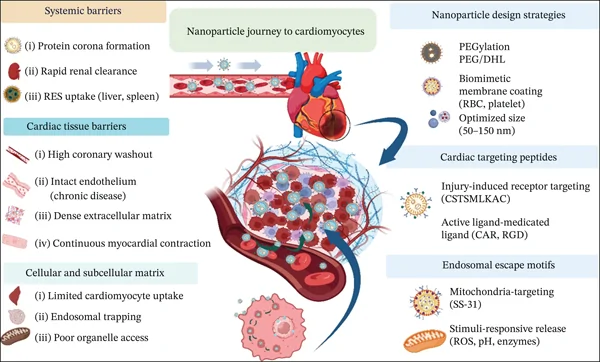
Biological barriers to cardiomyocyte‐targeted nanoparticle delivery and rational design strategies to overcome them. Schematic illustrating barriers to cardiomyocyte nanoparticle delivery and strategies to overcome them. System‐level barriers include protein‐corona formation, renal clearance, and RES uptake. Tissue‐level barriers include high coronary flow, intact endothelium, dense ECM, and myocardial contraction. Cellular barriers include limited uptake, endosomal trapping, and poor organelle access. Rational design strategies like PEGylation, biomimetic coatings, optimized size (50–150 nm), targeting peptides, active uptake, endosomal escape, organelle ligands (TPP, SS31), and stimuli‐responsive release address these barriers, enabling hierarchical tissue‐to‐cell‐to‐organelle targeting.

### 7.1. Small Molecules

Small molecules play a crucial role in many therapeutics, including cardiovascular therapeutics, because they can modulate intracellular pathways, inhibit enzyme activity, alter ion channel function, or bind receptors on cardiomyocytes. The low molecular weight of these small molecules enables efficient transmembrane transport, allowing them to access intracellular targets. However, poor aqueous solubility, metabolic instability, and short half‐life limit the therapeutic index in CVD therapy. NP carriers such as liposomes, PLGA NPs, and dendrimers can enhance solubility, prolong circulation, and improve pharmacokinetics while enabling controlled or triggered release. These platforms also permit surface functionalization with cardiomyocyte‐targeting peptides or ischemia‐sensitive ligands, increasing myocardial accumulation and limiting off‐target deposition [228–230].

Selection of NPs for small molecules is importantly influenced by the disease environment. In MI, vascular permeability permits passive accumulation of PLGA or liposomal carriers (50–200 nm), while in HF, the fibrotic tissue barrier and reduced perfusion limit accumulation of small particles with active targeting ligands [130]. In atherosclerosis, NPs encapsulating small‐molecule carriers should cross the thickened intima to reach macrophage‐rich regions of the plaques, favoring particles < 100 nm with surface charge optimized to avoid rapid RES clearance [109].

Preclinical studies show that encapsulated small molecules can reduce infarct size, improve cardiac function, and attenuate oxidative or inflammatory injury far more effectively than free drugs. For example, PLGA‐encapsulated irbesartan has shown efficacy in reducing inflammatory monocyte infiltration and infarct size in mice. Similarly, liposomal glucocorticoids have shown anti‐inflammatory effects in atherosclerosis [231]. Yet most of these findings derive from acute injury models in healthy rodents. Trials comparing NP‐bound versus optimized free‐drug formulations are limited, and the influence of comorbidities (diabetes, obesity, aging) on drug release and targeting remains underexplored. Understanding how disease‐specific pH, enzyme activity, and inflammatory conditions change during small‐molecule release from particles requires deeper mechanistic studies.

### 7.2. Proteins

Proteins also play a therapeutic role in treating heart diseases. Proteins such as growth factors, cytokines, enzymes, and antibodies play a central role in modulating post‐infarction remodeling, angiogenesis, and the inflammatory response following cardiac injury [232]. However, these proteins pose challenges because they are susceptible to enzymatic degradation and proteolysis, are rapidly cleared, and often fail to cross cellular membranes. Unfortunately, no single effective method for protein delivery has proven successful in addressing these concerns; however, numerous approaches using NP delivery systems have been developed to encapsulate or conjugate proteins to the NP surface, thereby improving stability and bioavailability. Therefore, NP delivery systems address many of these limitations by encapsulation or surface conjugation, thereby enhancing stability, preventing enzymatic degradation, and enabling controlled or stimuli‐responsive release [228, 230, 233].

The choice of NP platform for protein delivery must account for the disease microenvironment’s unique characteristics. In MI, the inflammatory milieu with elevated proteases requires carriers that protect proteins from enzymatic degradation while enabling rapid release during the acute phase. In HF, sustained protein delivery (e.g., growth factors for angiogenesis) requires carriers with extended‐release profiles and the ability to penetrate fibrotic tissue. The acidic pH and oxidative stress in the ischemic or failing heart can be exploited for stimuli‐responsive release of protein therapeutics using pH‐sensitive polymers or ROS‐cleavable linkers [91, 234].

### 7.3. Genes

Gene therapy offers powerful opportunities to correct genetic defects, control pathological gene expression, or promote cardiac regeneration by delivering DNA, RNA, or mRNA systemically or locally. Targeted delivery to cardiomyocytes could offer a therapeutic approach for certain heart diseases. However, nucleic acids degrade rapidly in biological fluids, exhibit poor cellular uptake, and risk innate immune activation. NPs, particularly LNPs, PNPs, and membrane‐coated carriers, provide critical protection from nuclease degradation and enable more efficient endosomal escape [238, 239]. Recent advances in LNPs have improved delivery of RNA to the heart [26]. Optimized LNPs can deliver mRNA to damaged heart tissue, reduce liver uptake, and increase cardiac gene expression. New high‐throughput screening methods also help identify the most effective LNP formulations for cardiac mRNA delivery [240].

Recent studies demonstrate successful transfection of cardiomyocytes using NP systems, opening avenues for treating inherited cardiomyopathies or reprogramming fibroblasts into cardiomyocyte‐like cells [43]. For example, CRISPR‐Cas9 delivery using LNPs has shown potential for correcting genetic mutations in Transthyretin Amyloidosis‐related cardiomyopathies [241]. Recent advances illustrate increasing feasibility of cardiac RNA delivery. Evers et al. demonstrated systemic LNP‐mediated modified mRNA delivery to damaged myocardium, while Shuvaev et al. reported systemic biotherapeutic RNA delivery that transiently modulated myocardial function [111, 242]. More recently, Neiman et al. developed a cardiac microphysiological screening system that predicts in vivo heart transfection efficiency of LNP–mRNA formulations [240]. Together, these studies highlight both the progress in cardiac RNA delivery and the need for improved screening strategies to identify formulations with genuine myocardial tropism.

Nonetheless, most data remain in the early stages. Key questions include whether NPs can deliver genetic cargo at clinically meaningful efficiency in the heterogeneous, high‐blood‐flow environment of the human heart, and whether chronic gene modulation using repeated NP dosing will lead to sustained benefit without immunologic or genomic toxicity. Comparisons with viral delivery vectors, currently the clinical gold standard, are also lacking. Repeated use of LNPs may trigger proinflammatory immune responses and unintended clearance through anti‐PEG antibodies, which could reduce the effectiveness and safety of long‐term gene therapy.

## 8. Drug Release Patterns

NPs enable efficient delivery of diverse therapeutic cargos, including small molecules, proteins, and genes, through controlled and stimuli‐responsive release mechanisms (Figure 5). The therapeutic performance of a NP platform is critically shaped by its drug release profile, which determines when, where, and how efficiently the payload becomes bioavailable within cardiac tissue. In cardiomyocyte‐targeted therapy, timing, localization, and precision are essential to limit off‐target toxicity and enhance myocardial uptake; therefore, engineered release mechanisms have become especially important. NPs can be tailored for immediate, sustained, or stimuli‐responsive release, enabling controlled exposure aligned with disease pathology, such as the acute burst required after MI, or the prolonged delivery needed for chronic HF. Understanding and optimizing these release patterns is therefore central to designing practical and clinically translatable cardiac nanotherapeutics. Importantly, the effectiveness of these release strategies depends heavily on disease‐specific micro‐environmental cues, further emphasizing the need for biologically informed NP design.

**Figure 5:**
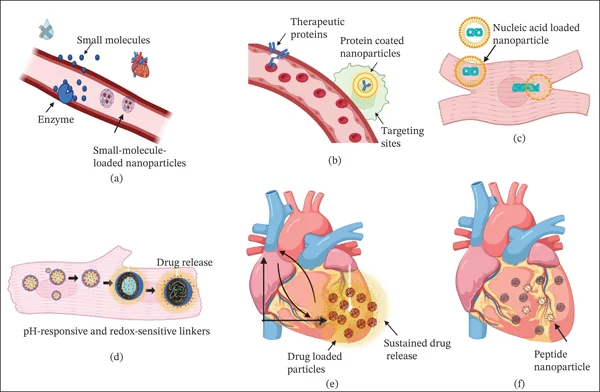
Nanoparticle‐mediated delivery of therapeutic cargo and controlled release strategies for cardiac applications. (a) Encapsulation of small molecules improves solubility, stability, and intracellular delivery. (b) Nanoparticle encapsulation or surface conjugation protects therapeutic proteins from enzymatic degradation and enables controlled release. (c) Nanocarriers protect nucleic acids from extracellular degradation and facilitate cellular uptake, endosomal escape, and intracellular cargo release. (d) Intracellular release following nanoparticle endocytosis and endosomal escape enables cytoplasmic delivery of therapeutic cargo. (e) Sustained‐release systems provide prolonged local drug exposure within injured myocardium. (f) Ligand‐functionalized nanoparticles enhance preferential accumulation and receptor‐mediated delivery at diseased cardiac tissue compared with untargeted nanoparticles. Collectively, these strategies improve cargo stability, spatial and temporal control of release, and therapeutic delivery to cardiac tissue.

### 8.1. Precision Intracellular Release

For most cardiac therapeutics, intracellular delivery is essential, and NPs facilitate endocytosis and release their contents in response to intracellular triggers such as acidic pH, elevated ROS, reduced cytoplasmic pH, or enzymatic cues. pH‐sensitive or redox‐sensitive linkers could be employed to ensure that drugs are preferentially released within cardiomyocytes, thereby enhancing therapeutic potency while minimizing systemic toxicity [230, 243].

Dual‐targeting NPs improve drug delivery by combining heart cell‐targeting with organelle‐targeting molecules, such as mitochondrial or nuclear targeting agents. These multilevel systems help NPs overcome biological barriers, improve cardiomyocyte‐specific uptake, and deliver the drug to the desired intracellular location [244].

Intracellular delivery has proven crucial in gene editing (e.g., CRISPR), mitochondrial therapeutics, and protective protein delivery after ischemia–reperfusion [245]. For mitochondrial targeting, TPP helps NPs accumulate in mitochondria, while SS31 protects the inner mitochondrial membrane and reduces oxidative damage. Combining TPP or SS31 with cardiomyocyte‐targeting ligands is a promising strategy for treating mitochondrial dysfunction in heart diseases [244]. Although preclinical studies consistently demonstrate enhanced cell survival and improved cardiac function, quantifying intracellular payload distribution remains technically challenging. Advanced imaging, single‐cell pharmacokinetics, and organelle‐level localization studies are needed to understand how NPs traverse intracellular compartments and how these pathways differ in disease states.

### 8.2. Slow Release

Slow or sustained‐release NPs provide extended therapeutic concentrations with fewer administrations, improving patient adherence and supporting long‐term myocardial repair. PLGA NPs and hydrogel–NP composites offer controlled release over days to weeks via degradation‐ or diffusion‐based mechanisms. Such systems have shown benefits in chronic cardiac diseases and in sustaining reparative activity after MI, including enhanced angiogenesis, reduced fibrosis, and improved cardiomyocyte survival [246–248]. In cardiac therapy, this approach offers the greatest value in chronic disease or for supporting tissue repair after an insult like MI.

Slow‐release drug delivery should be designed according to the disease. In HF, smaller NPs with longer circulation are preferred because the scar tissue and poor blood flow limit the large hydrogel systems. In atherosclerosis, sustained drug release within plaques may reduce frequent dosing, but plaque structure is different, and drug penetration must be considered [249].Notably, sustained delivery of vitamin D‐based anti‐inflammatory NPs through a one‐shot injectable hydrogel has been shown to induce regulatory T cells in the atherosclerotic lesions [250].

Although slow release offers these benefits, in vivo release kinetics remain difficult to predict because of enzymatic activity, myocardial perfusion, pH, and inflammatory conditions vary. Diseases like diabetes and obesity can change how polymer‐based drug carriers break down in the body, which can affect drug release. Slow release can also result in chronically low drug levels insufficient for therapeutic action. Additionally, hydrogels and solid polymer materials may alter local tissue stiffness, potentially influencing cardiac electrophysiology and remodeling if administered in excess, an area requiring critical investigation.

### 8.3. Release at the Disease Site

Targeted delivery strategies aim to increase drug concentration at the diseased myocardium while minimizing systemic toxicity. This can be achieved by functionalizing NPs with ligands, peptides, aptamers, or antibodies that bind cell‐specific receptors, and selective accumulation of therapeutic molecules in cardiomyocytes, endothelial cells, and activated fibroblasts [248, 251, 252]. For example, platelet‐membrane‐coated NPs and ischemia‐targeting peptides significantly enhance deposition in infarcted myocardium following intravenous injection [253]. Targeted delivery strategies consistently outperform passive targeting alone in preclinical studies. Yet significant gaps remain, including receptor expression that varies dynamically across disease stages; ligand density and orientation, which strongly affect binding but are poorly standardized; and the complex, large‐scale manufacturing of ligand‐functionalized NPs. Moreover, clinical translation will require validated biomarkers of target expression and standardized assays to assess binding specificity. Targeting ligands should be selected based on the disease. CTP and AT1 receptor ligands are suitable for MI, NPR ligands for HF, and mitochondrial targeting ligands for DOX‐induced cardiotoxicity. Choosing ligands based on disease‐specific receptor expression can improve targeted drug delivery and clinical translation [254].

## 9. Administration Methods and Safety Considerations

NP‐based delivery of therapeutics to cardiomyocytes can be achieved through various routes of administration, each with its own advantages and disadvantages. The most common route is intravenous infusion, which enables rapid systemic distribution and leverages the EPR effect to target the heart. Cardiac‐targeting peptide surface modification significantly improves heart‐tissue retention and specificity. Intracoronary infusion is another access route that can achieve local concentration but is more invasive. Inhalation has also emerged as a noninvasive route for repeated cardiac nanomedicine delivery, particularly in chronic HF, although pulmonary deposition and clearance remain important determinants of cardiac bioavailability [196]. Oral formulations are in the pipeline, pending noninvasive availability, but additional evidence is needed to assess absorption and biodistribution. Lastly, delivery must balance efficacy, safety, and the clinical context of cardiac disease [229, 251, 252].

The cardiac system is sensitive to NP‐induced toxicity due to the heart’s continuous electrical and mechanical activity, limited regenerative capacity, and dependence on mitochondrial function [36]. Importantly, safety comes first in NP‐mediated drug delivery to cardiomyocytes because physicochemical properties such as size, surface, and composition can affect biological interactions and even induce nanotoxicity, oxidative stress, inflammation, and vascular damage.

Biocompatibility and biodegradability, achieved using lipids, polymers, and peptides, are required to prevent immunogenicity and long‐term accumulation. Surface functionalization, e.g., PEGylation or biomimetic coating, also increases circulation time and reduces immune recognition, whereas the PEG dilemma should be considered. Protein‐ and peptide‐grafted NPs offer additional safety advantages, as they are naturally derived and enable site‐specific drug delivery, minimizing cytotoxicity and enhancing cardiomyocyte viability.

Long‐term safety evaluation is essential because many CVDs require chronic treatment and repeated NP administration [255]. Preclinical studies should be conducted with caution to assess acute and chronic toxicity, immunogenicity, biodistribution, and off‐target effects before clinical application. For diseases such as HF and atherosclerosis, repeated NP administration requires a clear understanding of long‐term biodistribution, accumulation in the RES, and the risk of delayed toxicity. Biodegradable NPs, such as those made from PLGA or lipids, are generally preferred for chronic therapy because they clear more easily from the body and carry a lower risk of cumulative toxicity than nonbiodegradable materials, such as metals, carbon‐based NPs, or some dendrimers [256]. Lastly, one must consider extensive long‐term safety data to ensure safe NP delivery in cardiac therapy [171, 257, 258].

Safety evaluation should also be specific to CVD. In MI, NPs should not exacerbate reperfusion injury or interfere with physiologic resolution of inflammation. In HF, they should not depress myocardial contractility or increase arrhythmia risk. In atherosclerosis, they should not accelerate plaque progression or destabilize vulnerable plaques. In DOX‐induced cardiotoxicity, the NPs should not increase further oxidative stress or cardiac injury [259, 260].

## 10. Regulatory Requirements and Translational Considerations

NP‐mediated drug delivery presents complex regulatory challenges because the therapeutic performance and safety of nanomedicines depend not only on the active pharmaceutical ingredient but also on the carrier’s physicochemical characteristics. Regulatory agencies such as the FDA and EMA therefore require detailed characterization of NP composition, size and size distribution, surface charge, morphology, drug‐loading efficiency, release kinetics, stability, and manufacturing reproducibility, together with robust preclinical and clinical evaluation of safety, efficacy, and quality. For actively targeted cardiac nanomedicines, additional characterization of targeting‐ligand identity, density, orientation, conjugation stability, and biological activity is important because relatively small changes in these parameters can alter biodistribution and target‐cell uptake.

Cardiovascular applications introduce additional safety considerations because NPs are administered directly into a highly perfused system and may interact with blood components, vascular endothelium, and cardiac tissue. Preclinical evaluation should therefore extend beyond conventional cytotoxicity to include hemocompatibility, complement activation, thrombogenicity, immunogenicity, cardiac electrophysiology and arrhythmogenic potential, and functional contractility assays, myocardial accumulation, and quantitative biodistribution to major off‐target organs such as the liver, spleen, kidney, and lung. For cardiomyocyte‐targeted systems, whole‐heart accumulation alone is insufficient to demonstrate specificity; cell‐level uptake should be established and, where relevant, intracellular or organelle localization.

Another major translational challenge is the potential difference between laboratory‐scale and GMP‐scale NP production. Changes in particle size, polydispersity, surface ligand density, encapsulation efficiency, or drug‐release characteristics during scale‐up can substantially affect pharmacokinetics and therapeutic efficacy [261]. Of note, shelf‐life stability should be of an important concern as NP aggregation can lead to loss of its structural integrity and premature drug release inside the vials. Specifically, this is a very commonly seen issue with NPs containing active ligands on their surface. Establishing critical quality attributes (CQAs), batch‐to‐batch reproducibility, validated sterilization/aseptic‐processing approaches and sterility assurance, storage stability, and quality‐by‐design principles is therefore essential for clinical translation. This challenge may be even greater for complex dual‐targeted, stimuli‐responsive, hybrid, and extracellular‐vesicle‐based systems because each additional functional component introduces another source of manufacturing and regulatory variability.

The novelty of many nanomaterials also warrants careful assessment of their long‐term fate, biodegradation, bioaccumulation, and off‐target toxicity. These issues are particularly important for chronic CVDs such as HF, where repeated administration may be required, in contrast to short‐term treatment following acute MI. Accordingly, one should consider the intended disease, route of administration, dosing frequency, duration of exposure, and whether the NP or its degradation products persist after the therapeutic cargo is released for the regulatory submissions.

For NP systems delivering gene‐editing payloads, genotoxicity represents an additional regulatory concern because nonspecific editing may introduce permanent genomic alterations. Safety evaluation should therefore assess on‐target and off‐target editing, unintended insertions or deletions, chromosomal rearrangements, and the persistence of gene‐editing components in target and off‐target tissues. Where applicable, studies should also evaluate DNA damage and genomic stability, particularly after repeated dosing or prolonged exposure. These assessments are especially important for cardiac‐targeted systems because nonspecific editing in cardiomyocytes or off‐target organs may result in persistent effects that cannot be reversed after NP clearance. In contrast, nonintegrating mRNA and siRNA therapeutics do not inherently modify genomic DNA; their safety evaluation should instead emphasize off‐target pharmacology, innate immune activation, persistence of the nucleic‐acid cargo, and potential toxicities associated with the carrier or repeated administration. Patient‐Specific Variability: Regulatory submissions should consider patient heterogeneity, including age, comorbidities, and concomitant medications, and regulators must account for these factors during evaluation [262].

## 11. Current Limitations, Unresolved Challenges, and Future Directions

NP‐based therapeutics for CVD are advancing toward integration with genetic and molecular technologies. CRISPR‐Cas9 platforms have shown potential for correcting pathogenic cardiac mutations, while siRNA and mRNA delivery systems offer opportunities for cardioprotection and regeneration [263, 264]. LNP‐based gene‐modulating approaches further highlight the potential for durable therapeutic interventions [265]. Theranostic platforms combining diagnosis and therapy are also emerging, including fibrin‐targeted NPs for thrombus imaging and treatment and iron oxide‐based systems for simultaneous imaging and therapeutic delivery [266, 267]. Engineered EVs, dual‐targeted nanocarriers, and disease‐responsive systems further expand this landscape by enabling tissue‐, cell‐, and intracellular‐level delivery.

Despite these advances, several major barriers continue to limit clinical translation. These include low cardiomyocyte‐specific delivery following systemic administration, rapid liver and spleen clearance, protein‐corona formation, immune recognition, heterogeneous cardiac disease microenvironments, and potential toxicity following repeated administration. NP accumulation in the heart is often modest, and many systems achieve only enhanced cardiac exposure rather than true cardiomyocyte specificity. Third, protein‐corona formation in vivo alters NP identity, affecting target efficiency and biodistribution in unpredictable ways. Additional, unresolved challenges include the lack of standardized methods for evaluating cardiac versus off‐target biodistribution, insufficient demonstration of precise cell‐specific uptake, limited long‐term safety data, and poor comparability across NP platforms. Furthermore, most efficacy studies rely on young, otherwise healthy rodents, which poorly represent the aging, comorbid cardiovascular population likely to receive these therapies.

Future research should therefore prioritize clinically relevant validation rather than increasing NP complexity alone. Studies should incorporate aged and comorbid animal models, large‐animal validation, clinically relevant dosing and administration routes, and direct comparisons with free drugs and standard‐of‐care therapies. For targeted systems, whole‐heart accumulation should not be considered sufficient evidence of cardiomyocyte specificity; cellular and, when claimed, organelle‐level localization should be experimentally demonstrated. Ultimately, successful clinical translation will require balancing targeting precision and therapeutic efficacy with long‐term safety, manufacturability, reproducibility, scalability, cost, and regulatory feasibility. Fourth, manufacturing challenges, including scalability, reproducibility, and regulatory compliance, hinder clinical development. Biologically derived platforms, particularly engineered EVs, may further complement synthetic NPs by combining endogenous intercellular communication mechanisms with therapeutic cargo delivery, although manufacturing standardization and reproducibility remain major barriers to translation.

In conclusion, while NP‐based drug delivery for CVDs has shown substantial promise in recent years, clinical translation remains distant from success. Rationally using FDA‐approved biomaterials and adapting design considerations to integrate disease biology and microenvironmental conditions may enable faster clinical translation. Addressing these limitations will require standardized evaluation frameworks, integration of disease biology into NP design, and rigorous validation in clinically relevant models.

**Nomenclature**
ACE: angiotensin‐converting enzyme
ANP: atrial natriuretic peptide
AMPK: AMP‐activated protein kinase
ARB: angiotensin receptor blocker
ASO: antisense oligonucleotide
BNP: brain natriuretic peptide
BiAbs: bispecific antibodies
CAD: coronary artery disease
CARPA: complement activation‐related pseudoallergy
cAMP: cyclic adenosine monophosphate
cGMP: cyclic guanosine monophosphate
CNTs: carbon nanotubes
CNP: C‐type natriuretic peptide
CRISPR: clustered regularly interspaced short palindromic repeats
CRP: C‐reactive protein
CTP: cardiac targeting peptide
CVD: cardiovascular disease
DALYs: disability‐adjusted life years
DAMPs: damage‐associated molecular patterns
DNA: deoxyribonucleic acid
DOX: doxorubicin
DZ: dexrazoxane
ECM: extracellular matrix
EF: ejection fraction
ELP: elastin‐like polypeptide
EPR: enhanced permeability and retention
ER: endoplasmic reticulum
ERK: extracellular signal‐regulated kinase
EVs: extracellular vesicles
FDA: Food and Drug Administration
G‐CSF: granulocyte colony‐stimulating factor
GBD: Global Burden of Disease
GC‐A: guanylate cyclase‐A receptor
GC‐B: guanylate cyclase‐B receptor
GMP: good manufacturing practice
GRAS: generally recognized as safe
HA: hyaluronic acid
HDL: high‐density lipoprotein
HF: heart failure
HFpEF: heart failure with preserved ejection fraction
HFrEF: heart failure with reduced ejection fraction
ICAM‐1: intercellular adhesion molecule‐1
IHD: ischemic heart disease
IM: intramuscular
INPs: inorganic nanoparticles
IR: ischemia–reperfusion
IV: intravenous
LDL: low‐density lipoprotein
LNPs: lipid nanoparticles
LV: left ventricle
mRNA: messenger RNA
miRNA: MicroRNA
MI: myocardial infarction
mPTP: mitochondrial permeability transition pore
MRI: magnetic resonance imaging
NLS: nuclear localization signal
NPR‐C: natriuretic peptide receptor‐C
NPs: nanoparticles
PAMAM: poly (amidoamine)
PEG: polyethylene glycol
PI3K: phosphoinositide 3‐kinase
PKG: protein kinase G
PLGA: poly (lactic‐co‐glycolic acid)
PLA: polylactic acid
PCL: polycaprolactone
PNPs: polymeric nanoparticles
RES: reticuloendothelial system
RGD: arginine‐glycine‐aspartic acid peptide
RNA: ribonucleic acid
ROS: reactive oxygen species
siRNA: small interfering RNA
SGLT2: sodium‐glucose cotransporter 2
SR‐A: scavenger receptor class A
SS‐31: Szeto–Schiller peptide 31
STAT3: signal transducer and activator of transcription 3
SLNs: solid‐lipid nanoparticles
SPIONs: superparamagnetic iron oxide nanoparticles
TGF‐*β*
: transforming growth factor‐beta
TfR1: Transferrin receptor 1
TPP: Triphenylphosphonium
VEGF: vascular endothelial growth factor
VEGFR2: vascular endothelial growth factor receptor 2
VCAM‐1: vascular cell adhesion molecule‐1
WHO: World Health Organization

## Author Contributions

Conceptualization: B.S., S.B., and S.V.; investigation: B.S. and S.V.; validation: B.S. and S.V.; visualization: B.S. and S.B.; writing – original draft: B.S.; writing, review and editing: B.S., S.B., and S.V.; supervision: S.B. and S.V.; funding acquisition: S.V.

## Funding

This work was supported in part by the National Heart, Lung, and Blood Institute of the National Institutes of Health under Award Number R01HL157335 and National Institutes of Health—National Institute of General Medical Sciences (NIH‐NIGMS) Tumor Microenvironment Centers of Biomedical Research Excellence (TME CoBRE) Grant [5P20GM121322].

## Ethics Statement

The authors have nothing to report.

## Consent

The authors have nothing to report.

## Conflicts of Interest

The authors declare no conflicts of interest.

## Data Availability

This article is a review manuscript and does not report original experimental data. This study generated or analyzed no new datasets.
